# W-AGRU-Net: a dual-stream attention framework for robust brain tumor segmentation in clinical MRI imaging

**DOI:** 10.1038/s41598-026-62348-y

**Published:** 2026-08-27

**Authors:** Marwa Abbas, Hussein Mogahed, Aziza Hussein, Ashraf Khalaf, Mohamed Mourad

**Affiliations:** 1 Computer and Systems Engineering Department, Faculty of Engineering, Minia University, Minia, 61111 Egypt; 2 Electrical and Computer Engineering Department, Faculty of Engineering, Effat University, Jeddah, 34689 Saudi Arabia; 3 Department of Electrical Engineering, Faculty of Engineering, Minia University, Minia, 61519 Egypt; 4 Space Communication Department, Faculty of Navigation Science and Space Technology, Beni-Suef University, Beni Suef, 62511 Egypt

**Keywords:** Brain tumor segmentation, Deep learning, U-Net, Attention mechanisms, Medical image analysis, MRI, Cancer, Computational biology and bioinformatics, Mathematics and computing, Medical research, Oncology

## Abstract

Segmenting brain tumors from MRI scans is a challenging aspect of medical image analysis because of anatomical complexity, ambiguous tumor boundaries, and variability of shape. In this paper, we propose W-AGRU-Net, a new dual-stream U-Net framework that combines W-Attention mechanisms with residual connections for strong and accurate glioma segmentation. Our framework uses two asymmetric streams that have pyramid-shaped dilated convolution schemes (PDCS) for hierarchical multi-scale context capturing. The framework also includes residual units that utilize Squeeze-and-Excitation (SE) blocks for channel-wise recalibration of features across multiple resolutions, thereby strengthening relevant tumor regions while reducing noise. The method is thoroughly evaluated on the TCIA LGG Segmentation and Figshare datasets, where it outperformed the state-of-the-art approaches. On the Figshare dataset, the approach achieved best-in-class metrics, including a registration Dice coefficient of **97.87%** and Jaccard index of **97.44%**, as well as 95.14% precision and 93.42% sensitivity. On the TCIA dataset, we achieved **94.0% Dice**, **99.9% pixel-level accuracy**, **89.29% Jaccard index**, and **90.01% sensitivity**, demonstrating improved performance over baseline methods. Concerning more recent state-of-the-art approaches with Dice scores of 92.0% (TCIA) and 96.9% (Figshare), our approach showed enhanced accuracy in brain tumor segmentation, and we expect our approach to work well for clinical brain imaging applications.

## Introduction

Brain tumors, an atypical multiplication of brain cells, are a widespread public health issue globally. The growing prevalence of malignant brain tumors has substantial consequences for both individual patients and society as a whole^1^. For example, according to the American Cancer Society’s Cancer Statistics Center, in the United States in 2019, there were an estimated 23,820 new brain tumors diagnosed and about 17,760 deaths^2^. Gliomas are the most common primary brain tumors. They are clinically classified as low-grade gliomas (LGGs) and high-grade gliomas (HGGs) based on the pathology.

Magnetic Resonance Imaging (MRI) is the most widely used modality of neuroimaging analysis mainly because of its excellent soft tissue contrast and anatomical/metabolic information on brain tumors^2,3^. In-depth anatomical and physiological details of MR images enable complete data exploration of tumor morphology, aid in clinical decision support for treatment planning, and serve as a baseline prior to therapeutic response monitoring. Therefore, MRI-based pattern recognition for brain tumor detection/delineation has been an emerging hotspot in the current state-of-the-art pipelines of both neuro-oncology and medical imaging analysis^3^.

Standard segmentation techniques such as region growing, edge detection, and thresholding rely on hand-crafted features and fail to capture tumor morphological heterogeneity, intensity variability, and indistinct tumor margins in brain MRI. Convolutional neural networks (CNNs) have revolutionized medical image analysis, but the traditional U-Net model^4^ have several shortcomings. These include overfitting with limited annotated data, limited contextual information due to localized receptive fields, and a lack of multi-scale representation for gliomas with different sizes and infiltration patterns.

To address these limitations, this paper proposes W-AGRU-Net, a unified dual-stream attention-guided residual framework for brain tumor segmentation. The proposed architecture integrates complementary local and global feature learning through attention-guided residual units, squeeze-and-excitation blocks, and pyramid-shaped dilated convolutions.

The primary objective of this work is not merely to combine existing architectural components, but to provide a unified and reproducible segmentation framework that jointly exploits complementary local texture information, global contextual dependencies, and cross-stream feature interaction. By integrating dual-stream feature learning, attention-guided residual units, squeeze-and-excitation blocks, and pyramid-shaped dilated convolutions within a single architecture, the proposed W-AGRU-Net addresses these complementary challenges simultaneously, leading to improved segmentation accuracy while maintaining computational efficiency.

**The main contributions of this paper are fourfold**:

(1) Unified Dual-Stream Attention-Residual Framework: We propose W-AGRU-Net, a unified dual-stream encoder-decoder architecture that jointly models complementary local and global contextual information through attention-guided residual learning, squeeze-and-excitation blocks, and pyramid-shaped dilated convolutions.
(2) Multi-scale Context Integration: We introduce a Pyramid-Shaped Dilated Convolution Scheme (PDCS) into the proposed architecture to overcome the limitations of standard convolutions when segmenting tumors with large variations in size. Asymmetric dilated convolutions are employed to capture rich multi-scale contextual information.
(3) Adaptive Feature Recalibration: Squeeze-and-Excitation (SE)-Augmented Residual Units improve model discriminability by recalibrating channel-wise features dynamically, highlighting task-relevant features across various resolutions.
(4) Comprehensive Experimental Validation: Extensive benchmarking and evaluation on the Figshare and TCIA low-grade glioma (LGG) datasets, including fair comparisons with re-implemented baseline models under identical experimental settings and recently published state-of-the-art methods, demonstrate the effectiveness of the proposed W-AGRU-Net.

The remainder of this paper is organized as follows: Sect.  2 reviews related work in deep learning for brain tumor segmentation. Section  3 details the W-AGRU-Net architecture. Sections  4 and 5 present experimental results and conclusions, respectively.

## Related work

### U-Net architecture and initial improvements

U-Net^4^ architecture has changed the field of medical image segmentation and consists of a U-shaped encoder-decoder architecture with skip connections used to maintain spatial information during the down-sampling process. This architecture has been the foundation for countless advancements in the area of medical image segmentation. Although the performance of U-Net was impressive, researchers and practitioners started to realize its challenges in segmenting complex tumor structures, which motivated improved versions of the architecture. The first-generation versions were mostly focused on tackling architectural challenges, particularly concerning gradient propagation and inadequate feature representations, which led to more comprehensive enhancements proposed in subsequent studies.

### Integration of attention mechanisms

Attention mechanisms have also been applied to brain tumor segmentation. Inspired by the idea of residual learning, Zhang et al.^5^ performed extensive ablation studies on the impact of the attention mechanism, specifically attention gates, on the task of brain tumor segmentation from MRI. The model AGRU-Net that they developed aimed to improve the segmentation of small brain tumors. The experimental results showed good results with Dice scores on the BraTS 2019 dataset of 0.87, 0.777, and 0.709 for whole tumor, core, and enhancing tumor, respectively. The same holds for the BraTS 2018 dataset, where the model achieved a score of 0.872, 0.808, and 0.772 for whole tumor, core, and enhancing tumor, respectively, again validating the success of attention approaches.

Saifullah et al.^6^ drew attention to the benefits of the attention mechanism and showed that the modified U-Net architecture achieved a DSC of 0.9216 and JI of 0.8556 on the more challenging BraTS 2021 benchmark. The results clearly show that using an attention-based architecture can benefit from fine-tuning the features used by the model, as well as aid in defining the borders around complex tumors with ambiguous boundaries. These results were further supported by the work of Abd-Ellah et al.^7^, who proposed a novel approach for automatic brain tumor imaging using a symmetrical U-Net-style architecture with cascaded deep networks to identify and classify tumors. The proposed method was shown to achieve exceptional diagnostic performance (98.99%) and a Dice score of 0.91 in segmentation tasks on the BraTS 2017 benchmark.

### Multi-scale processing and hybrid architectural innovations

Another major advance in the brain tumor segmentation research space is the development of multi-scale processing abilities and hybrid architectural designs. Aboelenein et al.^8^ introduced an innovation known as the Hybrid Two-Track Architecture. The hybrid architecture, HTTU-Net, achieved Dice scores of 0.865 for the tumor region, 0.808 for the core region, and 0.745 for the improvement region, demonstrating its effectiveness in brain tumor segmentation tasks using the BraTS’2018 datasets.

Metlek et al.^9^ employed multi-scale processing capability. This was the inspiration for the development of the Res-UNet+ model, specifically for medical imaging, to augment feature extraction, providing local and global features at varying scales. The authors sought to employ compressed convolution operations that operate at the target scale, incorporating deep contextual information while achieving similar performance with a reduced computational budget. Repeatability was performed on three datasets, and the Res-UNet+ model yielded a Dice score mean of 92.80% for enhanced tumors, 93.10% for nuclei, and 91.90% for total tumors, showing the multi-scale processing capability.

Elaborating on the concept of the design principles of hybrid architecture, Saifullah et al.^10^ exhibited the results of the performance of DeepLabV3 + based on ResNet50 fusion with CLAHE-HE hybrid as a preprocessing step for brain tumor segmentation. Results obtained by this method are 99.93% accuracy, 0.9690 Dice score, and 0.9404 Jaccard index, while the most significant gain in performance was seen across tested architectures SAGA, SA-GA, LinkNet, MAG-Net, Edge U-Net, SegNet, and Multi-class CNN, proving a very high value of using preprocessing and hybrid approaches for boosting the robustness of segmentation in medical applications.

More recently, Zaman et al.^11^ proposed MSCS-Net, a multi-scale contextual segmentation network that combines multi-scale attention, contextual attention, and spatial pyramid pooling to improve lesion segmentation, particularly in low-resolution MRI. Although the framework demonstrated strong performance across several volumetric datasets, it was primarily designed for acute ischemic stroke lesion segmentation rather than brain tumor segmentation.

### Optimization techniques and advanced learning methodologies

The marriage of metaheuristic optimization with more sophisticated learning strategies has led to significant gains in efficiency and performance characteristics of the models. Saifullah and Dreżewski^12^ offered a hybrid model based on the modified U-Net, enhanced with an image pre-processing phase guided by the Particle Swarm Optimization (PSO). Their novel solution used PSO to dynamically enhance the image, and produced excellent results on a large dataset of 3,064 MRI scans, achieving a Dice of 96.99% and a Jaccard index of 94.21%. Performance is validated by cross-comparison with several well-established methods, including U-Net, LinkNet, SegNet, Active Contour, and Fuzzy C-Means, for which the authors note that the applied optimization strategy, while significantly advantageous, offers limited ability to generalize across heterogeneous datasets and imposes computational requirements.

Representative of this new wave of optimization approaches is a recent study^13^ that investigated the convergence of deep learning models with metaheuristic optimization methods. A PSO-optimized U-Net model was implemented with the specific aim of optimizing key hyperparameters correlated closely to performance (learning rates, dropout rates, etc.). This particular optimization model resulted in impressive gains, scoring a DSC of 94.14% and a JI of 89.02%. This model generalizes well to different tumor types, performing well with Meningioma, Glioma, and Pituitary tumors. This highlights the new opportunities and immense potential for optimization-driven CNNs in the context of medical image segmentation.

In Transfer Learning^14^, introduced a deep residual transfer learning approach for brain tumor classification. The deep network for feature learning was created by combining ResNet-50 and a fully convolutional CNN using convolutional and identity blocks. The proposed method uses Gaussian filtering and data augmentation for preprocessing. Multimodal fusion and U-Net-based segmentation are used for regression and classification. The approach achieved Dice scores of 0.88 (ET), 0.97 (WT), and 0.90 (TC), and 99.94% accuracy, 98.92% sensitivity, and 99.94% precision, with effective performance at a low computational cost.

In addition to the above methods, Singh et al.^15^ Proposed a deep learning-based hybrid approach for brain tumor analysis using MRI. The tumors were classified with an accuracy of 98.89% by the 2D CNN. The hybrid approach uses a thresholding and graph-based combination to segment the tumors on a series of MRI scans. These experiments show the power of incorporating CNN-based classification into traditional segmentation pipelines.

### Transformer-based architectures and advanced fusion models

Transformer-based architectures have recently been introduced to address the limitations of convolutional neural networks (CNNs) in capturing long-range dependencies and global contextual information. While CNN-based models are effective in extracting local features, they are inherently constrained by limited receptive fields. On the other hand, Vision Transformers (ViTs) provide strong global modeling capabilities through self-attention but often lack fine-grained spatial detail, which is essential for precise medical image segmentation. These complementary characteristics have motivated the development of hybrid CNN–Transformer architectures. In order to capture global contextual dependencies, Chen et al.^16^ presented TransUNet, a hybrid CNN–Transformer architecture that combines a convolutional encoder with a Transformer-based module. While fine-grained spatial details are restored using a U-Net-style decoder with skip connections, the model tokenizes CNN feature maps and uses self-attention to encode long-range associations. The authors achieved state-of-the-art outcomes for medical images by combining the benefits of accurate localization with global context.

Since CNN models were shown to be incapable of encoding long-range relations, Hatamizadeh et al.^17^ introduced Swin UNETR, a segmentation model inspired by vision transformers. This model was proposed to help physicians identify the presence of malignant tumors and follow the changes in their development. It achieved a Dice score of 85.3%, 92.7%, and 87.6% for ET, WT, and TC, respectively, and set a new state of the art for segmentation tasks with transformers on medical images. In^18^, the authors proposed a hybrid CNN–Transformer architecture that further enhances feature representation by combining convolutional feature extraction with Transformer-based global reasoning. The model achieved Dice scores of 83.72% and 86.32% on the BraTS 2018 and BraTS 2019 datasets, respectively, confirming the effectiveness of hybrid designs in medical image segmentation.

Enhancing fusion approaches, Rasool et al.^19^ proposed the integration of ResNet U-Net architecture with Transformer blocks to accurately localize diseased regions and anatomical landmarks using MRI sequences. Leveraging its ability to capture local and global context, the model improved the understanding of spatial relationships in image data.

Another model, ExU-Trans^20^ It was a U-Net-Transformer hybrid designed to perform brain tumor segmentation. It was able to achieve the leading Dice scores on the BraTS 2020 and 2021 challenges of 0.962 (whole tumor), 0.941 (tumor), and 0.944 (tumor core) while maintaining the features of the whole tumor regarding the U-Net structure. This approach was the first of its kind to achieve high performance on the challenges while using Transformer-based methods.

Additionally, an advanced hybrid framework that combines Swin Transformer with 3D convolutional feature extraction and hierarchical attention mechanisms was proposed in Advancing autonomous brain tumor segmentation with SwinT-UNet and a hierarchical attention module^21^.The architecture uses progressive refinement and multi-scale feature fusion to improve local structural preservation and global context modeling. In comparison to current hybrid CNN–Transformer techniques, experimental results show significant generalization ability across BraTS datasets and enhanced segmentation robustness.

Despite these developments, transformer-based systems usually have a high computational cost and need huge annotated datasets to train well. On the other hand, the suggested W-AGRU-Net uses a dual-stream attention-based CNN design that does not rely on heavy transformer components in order to achieve a balance between segmentation accuracy and computational efficiency.

### Anatomical knowledge-guided and context-aware segmentation approaches

Recent improvements in brain lesion segmentation go beyond architectural improvements. A combination of anatomical knowledge and specific heuristics appears to yield improvements over strictly data-driven approaches. For segmentation of brain lesions, the construction and physiology of the brain, as lesions tend to occupy various regions of the brain, may also inform the approach in a way that improves the performance and accuracy of the model.

For instance, lesion segmentation has been dynamically guided in the case of the presence of pathological morphological symmetry between the cerebral hemispheres. Robert, et al.^22^, proposed a pathological asymmetry guided segmentation of the acute ischemic stroke (PAPL) model. The PAPL model implemented a contrastive approach to learning asymmetric pathologically changing regions and integrated knowledge of pathology at various stages of the crystallized human learning process. The model also utilized morphology of symmetry of adjacent slices to a segmentation model to guide the interpretation of the context to a segmentation model.

The phenomenon of asymmetry pathology in the cerebral hemispheres, implemented in the PAPL model, contrastive learning, and segmentation of lesions, was enhanced by a methodology that also utilized knowledge and data that were not classified (i.e., labeled). The model proposed by Rech et al.^23^ implemented a multi-grained contrastive learning methodology to guide the segmentation of brain lesions while also functioning to classify the time of occurrence of the brain lesion (i.e., the onset of the brain lesion) based on integrated modalities of MRI data that were of high and various (heterogeneous) quality.

Additionally, multimodal imaging fusion has been widely adopted to incorporate complementary physiological information. In^24^ Diffusion- and perfusion-weighted MRI modalities were combined to develop automatic segmentation–classification models for identifying stroke within clinically critical time windows. The proposed framework demonstrated strong performance in both lesion segmentation and early stroke detection, highlighting the effectiveness of multimodal feature fusion.

More recently, Zhang et al.^25^ proposed SGAFNet, a learnable sequence-guided adaptive fusion network for robust brain tumor segmentation in situations with incomplete MRI sequences. This framework incorporates a Sequence-Guided Weighted Average (SGWA) module for adaptive fusion of sequence features and a Sequence-Specific Attention (SSA) module that connects cross-sequence dependencies. Tested on BraTS2018 and BraTS2020, SGAFNet showed competitive results in incomplete scenarios. In contrast to W-AGRU-Net, which focuses on single-modal T1-weighted MRI, SGAFNet is tailored for multi-modal scenarios with potentially missing sequences.

These methods showcase the increasing tendency towards the inclusion of anatomical priors and domain knowledge in concert with deep learning methods. While the effectiveness of these methods is clear, they typically integrate additional design modules or specific modality information. In this regard, the proposed W-AGRU-Net is based on a fully data-driven architecture that employs attention features and multi-scale contextual representation.

### Efficiency-oriented architectures and cross-domain inspirations

The drive for computational efficiency without sacrificing performance has led to better optimized architectural designs. Based on our systematic review of encoder architectures^26^, where we extensively evaluated twelve pretrained CNNs within U-Net architectures for brain tumor segmentation, MobileNetV2 performed the best with 99% cross-validation accuracy and Dice coefficients of 90.3% for Whole Tumor, 86.07% for Tumor Core, and 81.93% for Enhancing Tumor, respectively, on BraTS 2019. Thus, this work places MobileNetV2 as the best encoder backbone with performance-balanced accuracy and computational efficiency.

In this work^27^ Combined EfficientNetV2 with the U-Net to improve the performance of the segmentation model in locating and measuring brain tumors. The goal is to enhance diagnostic accuracy, save labor and time, and improve healthcare procedures. The results show that EfficientNetV2 can achieve 0.0866 loss, 0.9977 accuracy, and a 0.9133 DSC score when used in combination with U-Net.

Outside the niche of medical imaging, elegant optimization and feature selection methods have been successfully rolled out in other research contexts that may serve as useful inspiration for improving segmentation networks. Naiem et al.^28^, for example, introduced an iterative feature selection-based method of defending against DDoS attacks in cloud environments. This details the fundamental logic of feature selection, as well as illustrates the application of feature selection for improving the robustness and accuracy of the model. While their emphasis was not specifically on medical imaging, their work highlights the universal significance of optimized feature selection methodologies—principles that directly parallel the requirements for improving segmentation networks such as the W-AGRU-Net proposed in our current research.

In our previous work^29^ For the segmentation and classification of brain tumors, we suggested an integrated hybrid strategy. We employed a dual-encoder U-Net design for segmentation, which outperformed traditional U-Net and U-Net++, achieving a Dice score of 95.27% and an IoU of 86.89% on the Figshare dataset. We used a fine-tuned EfficientNetV2 S for classification. By combining attention gates, residual SE units, and pyramid-shaped dilated convolutions inside a dual-stream architecture, the suggested W-AGRU-Net outperforms this technique in segmentation performance (97.87% Dice).

The Figshare brain tumor dataset has recently been adopted by several studies to evaluate advanced segmentation frameworks. Li et al.^30^ proposed a multitask learning framework with multiscale residual attention (MMRAN), achieving a Dice score of 80.03% and a Jaccard score of 84.38%. Preetha et al.^31^ introduced a multi-scale attention U-Net with an EfficientNetB4 encoder, achieving a Dice score of 93.39% and a Jaccard score of 87.95%. Abbas et al.^29^ proposed a dual-encoder U-Net architecture that achieved a Dice score of 95.27% and a Jaccard score of 86.89%. In contrast, the proposed W-AGRU-Net achieved a Dice score of 97.87% and a Jaccard score of 97.44% on the same Figshare dataset. This improvement can be attributed to the complementary integration of dual-stream feature learning, attention-guided residual units, squeeze-and-excitation blocks, and pyramid-shaped dilated convolutions within a unified framework, enabling more effective modeling of both local details and global contextual information than approaches relying on individual architectural enhancements.

Recently, Chen et al.^32^ proposed YOLO-LS, a lightweight segmentation framework based on YOLO11n-seg, designed for brain tumor MRI. It utilizes Shuffle Net V1 as its backbone, DySample for dynamic up-sampling, and a C3k2-PoolingFormer module for effective feature fusion. YOLO-LS, evaluated on the Figshare dataset, achieved a Dice coefficient of 0.91 ± 0.01, an HD95 of 4.35 ± 0.34 mm, and reduced computational cost to 8.1 GFLOPs, marking a 15.6% reduction from the baseline, alongside improved mAP50. Additionally, it performed well on an external Kaggle dataset with a Dice of 0.895, demonstrating its suitability for resource-constrained clinical settings.

Our extensive analysis of the evolution of architecture, starting from simple U-Net variants to more complex transformer models and efficient architectures, provides fundamental context and indicates the research gaps that a W-AGRU-Net framework is designed to address. By combining the advantages of attention mechanisms, residual and multi-scale techniques, and feature optimization, and addressing their drawbacks, the proposed framework aims to advance the current research on brain tumor segmentation in clinical practice.

## Methodology

### Overview

While multi-scale convolutions and residual learning have been widely adopted in U-Net variants to mitigate vanishing gradient issues (e.g., mResU-Net^33^, Attention-enhanced residual architectures have further improved feature selectivity^34^. Existing methods still lack an explicit and unified mechanism for jointly modeling local texture, global contextual dependencies, and cross-branch feature refinement within a fully reproducible framework. To address these limitations, the proposed W-AGRU-Net is formulated as a fully specified dual-stream encoder-decoder architecture designed for reproducible brain tumor segmentation from single-channel MRI inputs. The model takes a single-channel, normalized, non-augmented MRI slice as input.

1 $$\:\begin{array}{cccc}&\:X\in\:{\mathbb{R}}^{256\times\:256\times\:1},&\:&\:\end{array}$$

where all pixel intensities are scaled to the range $\:\left[0,1\right]$. The corresponding ground truth mask is

2 $$\:\begin{array}{cccc}&\:Y\in\:\{\mathrm{0,1}{\}}^{256\times\:256\times\:1},&\:&\:\end{array}$$

with 1 indicating tumor (foreground) and 0 indicating background. There are two asymmetric encoder-decoder streams (Branches A and B) that learn complementary feature representations. **Branch A employs low-dilation convolutions** to capture fine-grained details and spatial contextual information from the background and small tumor regions, whereas **Branch B captures the global contextual information of large tumor regions**. The backbone is an Attention-Gated Residual U-Net (AGRU-Net) enhanced with Squeeze-and-Excitation (SE) blocks for adaptive channel-wise recalibration.

**Fig. 1:**
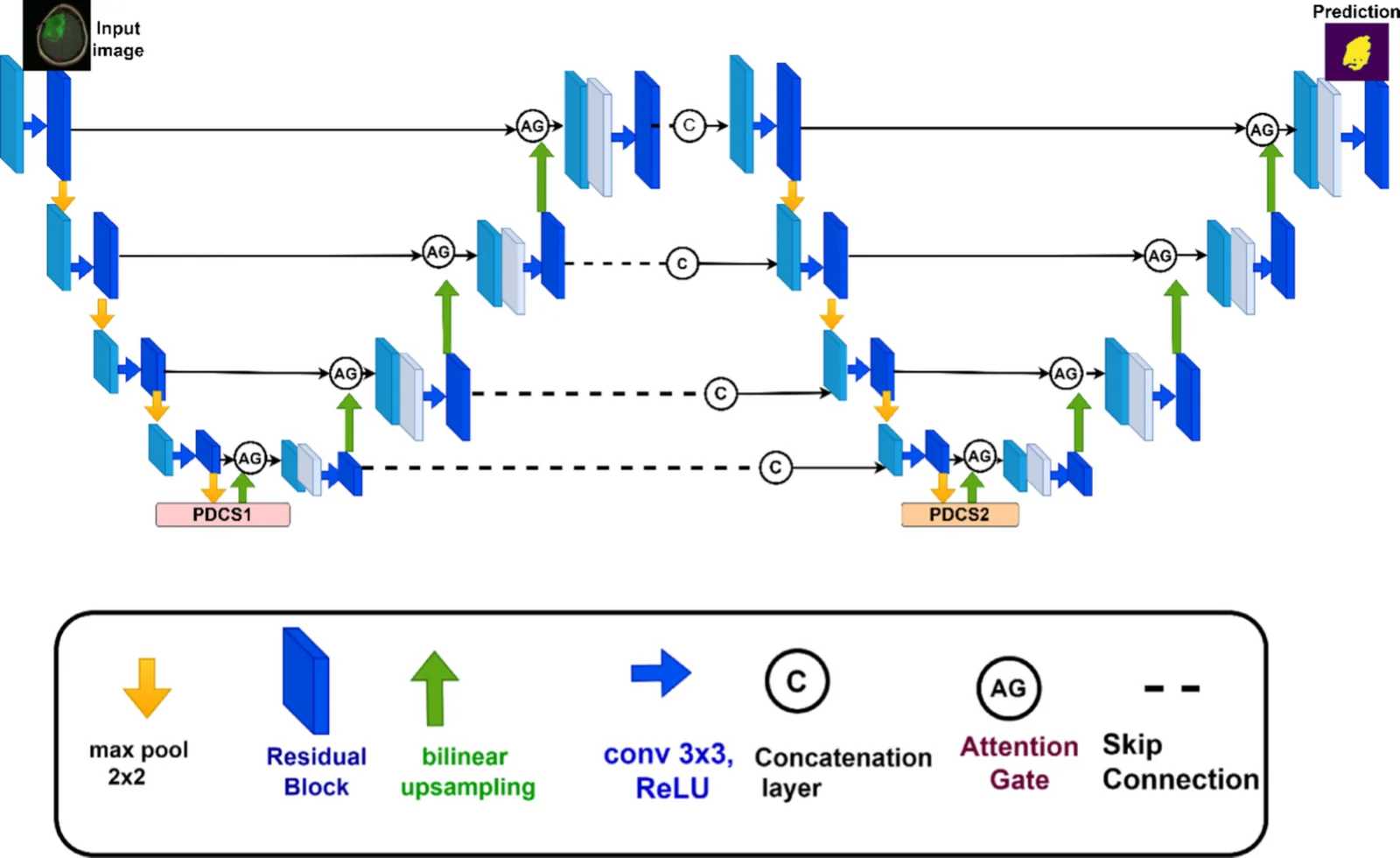
Overview of the proposed W-AGRU-Net architecture. The model follows a dual-stream encoder–decoder design with residual-SE blocks (blue), max-pooling (yellow arrows), bilinear up-sampling (green arrows), and skip connections (dashed lines). PDCS1 and PDCS2 modules capture multi-scale context, while Attention Gates (AG) and concatenation (C) (circular nodes) enhance feature relevance and fusion.

Figure 1 illustrates the complete architecture, where different colors and symbols represent specific operations: residual-SE feature extraction blocks are shown in blue, max-pooling operations in yellow, bilinear up-sampling in green, skip connections as dashed lines, and functional nodes such as attention gates (AG) and concatenation (C) are explicitly marked to clarify the information flow within the network.

To improve clarity and highlight the systematic evaluation strategy, Table 1 provides comprehensive definitions of all model abbreviations and descriptions. Each model variant was intentionally designed and implemented as part of an ablation study to quantitatively assess the contribution of individual architectural components.

**Table 1:** Summary of Proposed and Baseline Models with Abbreviations.

Model Name	Description
U-Net	Standard U-Net baseline.
W-U-Net	Two stacked U-Nets (W indicates “stacked” configuration)
AGU-Net	U-Net enhanced with Attention Gates (AG)
AGRU-Net	U-Net integrating Attention Gates with Residual Units.
W-AGU-Net	Two stacked AGU-Net architectures.
W-AGRU-Net (Proposed)	Final stacked AGRU-Net incorporating PDCS and SE modules.

### Attention-guided residual unit with squeeze-and-excitation

#### Structure of the enhanced residual block

Each basic residual block in the encoder contains two convolutional layers (Conv2D, kernel size $\:3\times\:3$), each followed by batch normalization (BN) and ReLU activation, a Squeeze-and-Excitation (SE) block that performs channel-wise feature recalibration, a skip connection that adds the input of the block to the output after the SE block and ReLU (element-wise addition, not concatenation), and finally an attention gate (AG) applied to the block output before it is passed to the next pooling layer or to the decoder. Let $\:\boldsymbol{x}$ be the input feature map to the block and $\:\boldsymbol{F}(\boldsymbol{x},\{{\boldsymbol{W}}_{\boldsymbol{i}}\left\}\right)$ the transformation performed by the two convolutional layers (including BN/ReLU). The output after the SE block and before the attention gate is:

3 $$\:\begin{array}{cccc}&\:{\boldsymbol{x}}_{\mathrm{res}}=\boldsymbol{x}+\mathrm{SE}\left(\boldsymbol{F}\right(\boldsymbol{x},\left\{{\boldsymbol{W}}_{\boldsymbol{i}}\right\}\left)\right),&\:&\:\end{array}$$

where $\:\mathrm{SE}(\cdot\:)$ denotes the channel recalibration function described below.

#### Squeeze-and-excitation (SE) block

**Fig. 2:**
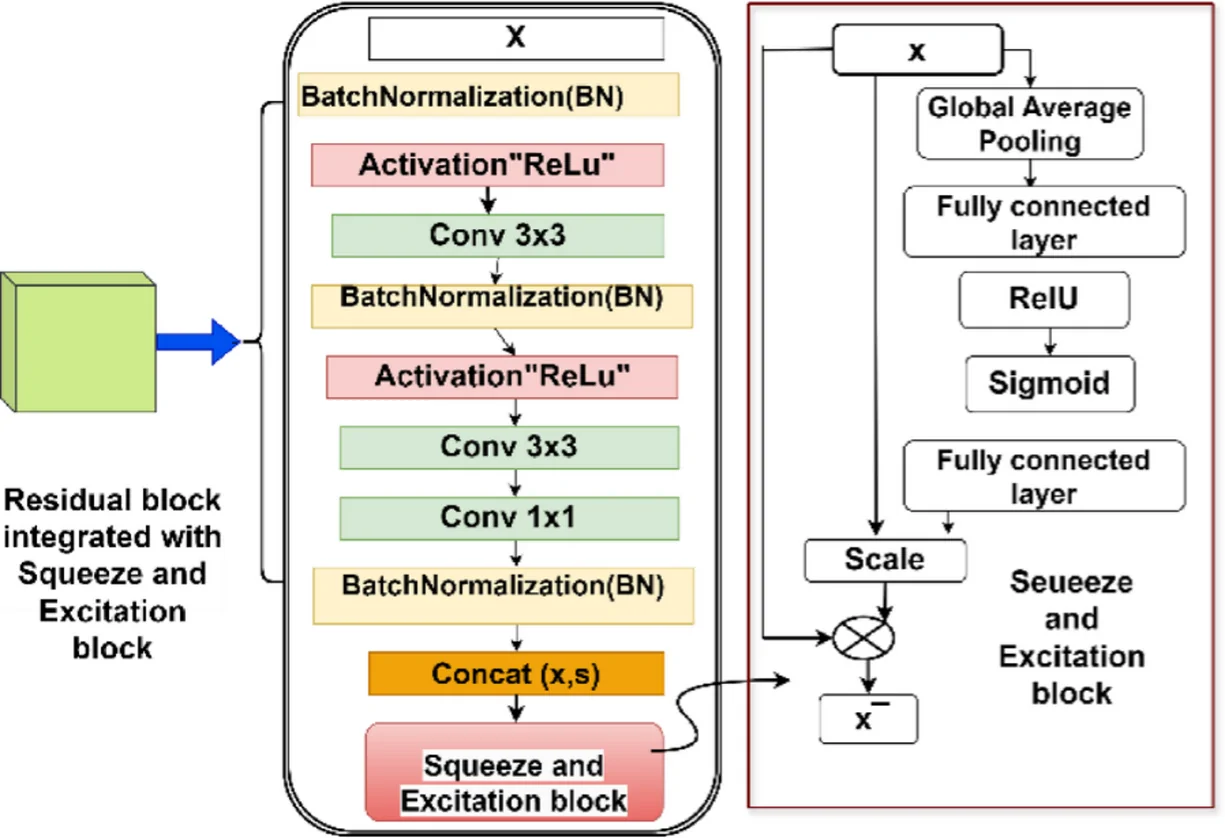
Expanding residual unit with excitation block and squeeze.

Let $\:\boldsymbol{U}\in\:{\mathbb{R}}^{\boldsymbol{H}\times\:\boldsymbol{W}\times\:\boldsymbol{C}}$ be the feature map produced by the last convolutional layer inside the block. The SE block, shown in Fig. 2, operates in three sequential steps. First, the squeeze operation applies global average pooling to compress each channel into a scalar:

4 $$\:\begin{array}{cccc}&\:{\boldsymbol{z}}_{\boldsymbol{c}}=\frac{1}{\boldsymbol{H}\times\:\boldsymbol{W}}\sum\:_{\boldsymbol{i}=1}^{\boldsymbol{H}}\sum\:_{\boldsymbol{j}=1}^{\boldsymbol{W}}{\boldsymbol{U}}_{\boldsymbol{c}}(\boldsymbol{i},\boldsymbol{j}),\boldsymbol{c}=1,2,\dots\:,\boldsymbol{C}.&\:&\:\end{array}$$

Second, the excitation operation passes the vector $\:\boldsymbol{z}$ through a small sub-network with two fully connected (FC) layers:

5 $$\:\begin{array}{cccc}&\:\boldsymbol{s}=\boldsymbol{\sigma\:}({\boldsymbol{W}}_{2}\cdot\:\boldsymbol{\delta\:}({\boldsymbol{W}}_{1}\boldsymbol{z}\left)\right),&\:&\:\end{array}$$

where $\:\boldsymbol{\delta\:}$ is ReLU, $\:\boldsymbol{\sigma\:}$ is the sigmoid function, $\:{\boldsymbol{W}}_{1}\in\:{\mathbb{R}}^{(\boldsymbol{C}/\boldsymbol{r})\times\:\boldsymbol{C}}$ and $\:{\boldsymbol{W}}_{2}\in\:{\mathbb{R}}^{\boldsymbol{C}\times\:(\boldsymbol{C}/\boldsymbol{r})}$ are the weight matrices, and $\:\boldsymbol{r}=16$ is the reduction ratio used throughout the proposed model. Third, the reweighting step scales each channel $\:{\boldsymbol{U}}_{\boldsymbol{c}}$ by the corresponding excitation value $\:{\boldsymbol{s}}_{\boldsymbol{c}}$:

6 $$\:\begin{array}{cccc}&\:{\stackrel{\sim}{\boldsymbol{U}}}_{\boldsymbol{c}}={\boldsymbol{s}}_{\boldsymbol{c}}\cdot\:{\boldsymbol{U}}_{\boldsymbol{c}}&\:&\:\end{array}$$

Unlike the original Squeeze-and-Excitation Network (SENet)^35^, which is typically used as a standalone channel -attention module in classification tasks, the proposed W-AGRU-Net integrates SE blocks within a residual encoder–decoder segmentation framework, jointly with attention gates and residual learning, to enhance both channel-wise importance and spatial tumor localization.

#### Attention gate (AG) – complete formulation

The attention gate receives two feature maps as input: encoder features $\:\boldsymbol{x}$ from the same level (skip connection) and a gating signal $\:\boldsymbol{g}$ from the decoder (up-sampled feature map from a coarser level). Let $\:{\boldsymbol{\sigma\:}}_{1}=\mathrm{ReLU}$ and $\:{\boldsymbol{\sigma\:}}_{2}=\mathrm{sigmoid}$. First, two linear transformations (implemented as $\:1\times\:1$ convolutions) Bring both inputs to the same number of internal channels $\:{\boldsymbol{F}}_{\mathrm{int}}=32$ (fixed across all attention levels):

7 $$\:\begin{array}{cccc}&\:\boldsymbol{\theta\:}={\boldsymbol{\sigma\:}}_{1}({\boldsymbol{W}}_{\boldsymbol{x}}^{\boldsymbol{T}}\boldsymbol{x}+{\boldsymbol{W}}_{\boldsymbol{g}}^{\boldsymbol{T}}\boldsymbol{g}+{\boldsymbol{b}}_{\boldsymbol{g}}),&\:&\:\end{array}$$

where $\:{\boldsymbol{W}}_{\boldsymbol{x}},{\boldsymbol{W}}_{\boldsymbol{g}}$ are the weight matrices of the $\:1\times\:1$ convolutions. Then a third $\:1\times\:1$ convolution (weights $\:\boldsymbol{\psi\:}$) reduces the channel dimension to one:

8 $$\:\begin{array}{cccc}&\:\boldsymbol{\alpha\:}={\boldsymbol{\sigma\:}}_{2}({\boldsymbol{\psi\:}}^{\boldsymbol{T}}\boldsymbol{\theta\:}+{\boldsymbol{b}}_{\boldsymbol{\psi\:}}).&\:&\:\end{array}$$

The coefficient $\:\boldsymbol{\alpha\:}\in\:[0,1{]}^{\boldsymbol{H}\times\:\boldsymbol{W}}$ This is the attention map. The final attended feature map is obtained by element-wise multiplication:

9 $$\:\begin{array}{cccc}&\:\widehat{\boldsymbol{x}}=\boldsymbol{\alpha\:}\odot\:\boldsymbol{x},&\:&\:\end{array}$$

and and $\:\widehat{x}$ is then concatenated with the up-sampled decoder features to guide the segmentation process. This completes the formulation of the proposed attention-guided residual unit.

### Pyramid-shaped dilated convolution scheme (PDCS)

**Fig. 3:**
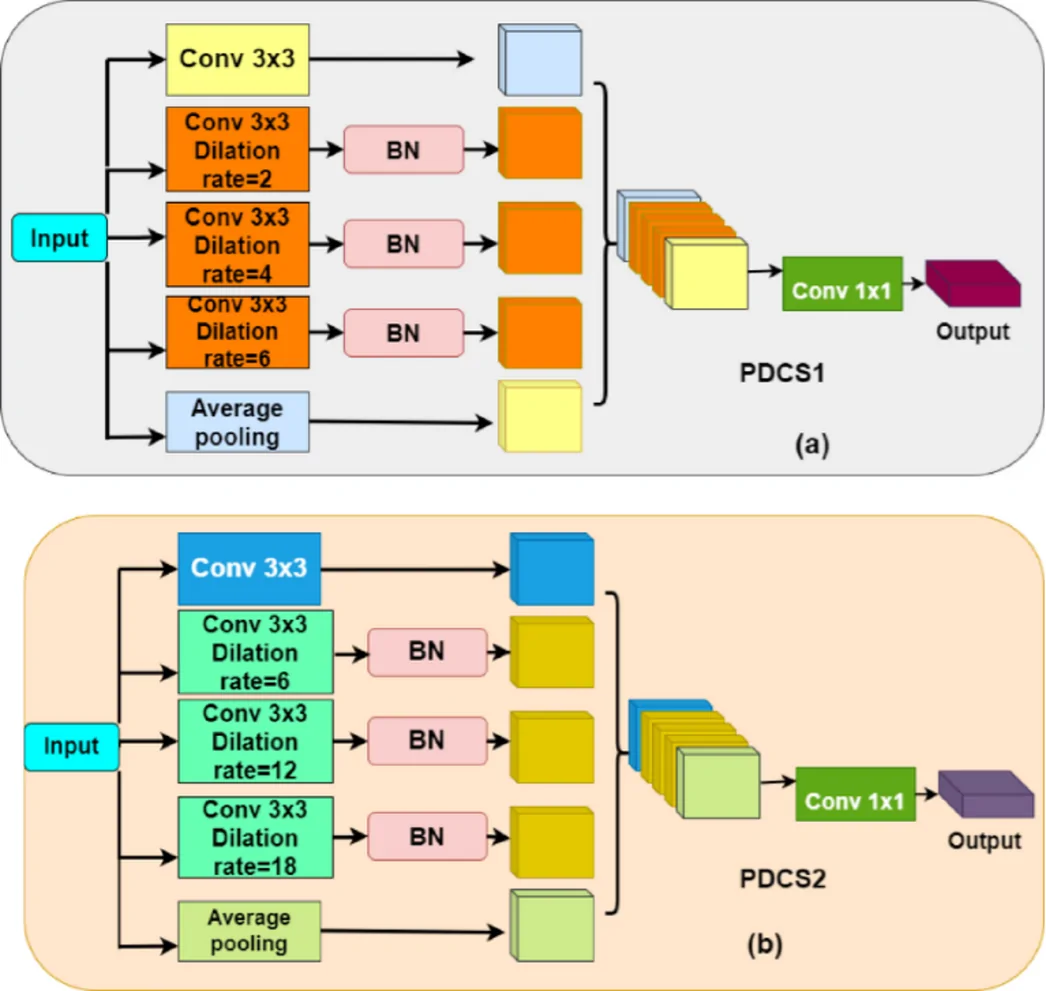
The pyramid-shaped dilated convolution schemes (PDCS) integrated within the dual-stream W-AGRU-Net framework: (**a**) PDCS1 with dilation rates {2, 4, 6}, (**b**) PDCS2 with dilation rates {6, 12, 18}. These modules enhance multi-scale contextual feature extraction.

Standard convolutions have fixed receptive fields and therefore cannot effectively capture multi-scale contextual information. We introduce two pyramid-shaped dilated convolution modules, PDCS1 (low dilation rates) and PDCS2 (high dilation rates), as illustrated in Fig. 3.

#### Dilated convolution (atrous convolution)

The dilated convolution operation with a dilation rate $\:\boldsymbol{r}$ is defined as:

10 $$\:\begin{array}{cccc}&\:\boldsymbol{y}[\boldsymbol{i},\boldsymbol{j}]=\sum\limits_{\boldsymbol{m}=0}^{\boldsymbol{k}-1}\sum\limits_{\boldsymbol{n}=0}^{\boldsymbol{k}-1}\boldsymbol{X}[\boldsymbol{i}+\boldsymbol{r}\cdot\:\boldsymbol{m},{\hspace{0.25em}\hspace{0.05em}}\boldsymbol{j}+\boldsymbol{r}\cdot\:\boldsymbol{n}]\cdot\:\boldsymbol{w}[\boldsymbol{m},\boldsymbol{n}],&\:&\:\end{array}$$

where $\:\boldsymbol{k}$ is the kernel size (here $\:\boldsymbol{k}=3$), and $\:\boldsymbol{r}$ controls the spacing between kernel elements. This expands the receptive field without increasing the number of parameters.

#### PDCS1 – low dilation rates {2,4,6}

For each dilation rate $\:\boldsymbol{r}\in\:\{2,4,6\}$, a dilated convolution followed by batch normalization (BN) and ReLU is applied:

11 $$\:\begin{array}{cccc}&\:{\boldsymbol{F}}_{\boldsymbol{r}}=\mathrm{ReLU}\left(\mathrm{BN}\right({\mathrm{Conv}}_{3\times\:3}(\boldsymbol{X},\boldsymbol{r})\left)\right),\boldsymbol{r}\in\:\{2,4,6\}.&\:&\:\end{array}$$

The three parallel outputs are then concatenated along the channel axis. A $\:1\times\:1$ convolution reduces the channel dimension to produce the final PDCS1 feature map:

12 $$\:\begin{array}{cccc}&\:{\boldsymbol{F}}_{\mathrm{PDCS1}}={\mathrm{Conv}}_{1\times\:1}\left(\right[{\boldsymbol{F}}_{2},{\boldsymbol{F}}_{4},{\boldsymbol{F}}_{6}\left]\right),&\:&\:\end{array}$$

where $\:\left[\cdot\:,\cdot\:,\cdot\:\right]$ denotes channel-wise concatenation.

#### PDCS2 – high dilation rates {6,12,18}

For efficiency, the high-dilation branch uses depthwise separable convolutions^30^. For each $\:\boldsymbol{r}\in\:\{6,12,18\}$:

13 $$\:\begin{array}{cccc}&\:{\boldsymbol{G}}_{\boldsymbol{r}}=\mathrm{DepthwiseSepConv}\left({\mathrm{Conv}}_{3\times\:3}\right(\boldsymbol{X},\boldsymbol{r}\left)\right),&\:&\:\end{array}$$

where $\:\mathrm{DepthwiseSepConv}(\cdot\:)$ First applies a depthwise convolution followed by a pointwise convolution^30^. The three branches are concatenated and passed through a $\:1\times\:1$ convolution:

14 $$\:\begin{array}{cccc}&\:{\boldsymbol{F}}_{\mathrm{PDCS2}}={\mathrm{Conv}}_{1\times\:1}\left(\right[{\boldsymbol{G}}_{6},{\boldsymbol{G}}_{12},{\boldsymbol{G}}_{18}\left]\right).&\:&\:\end{array}$$

PDCS1 is added in front of the 4th stage of the encoder (for finer details of the structure), and PDCS2 is added in front of the bottleneck (to capture the global context). As illustrated with respect to Fig. 3, the different dilation rates allow the network to learn a powerful multi-scale representation, which is important for precise brain tumor segmentation.

### Detailed architecture and quantitative specification

The proposed W-AGRU-Net follows a dual-stream encoder–decoder structure. Each encoder stage consists of two residual-SE blocks (as described in Sect.  3.2), followed by a $\:2\times\:2$ max-pooling operation for down-sampling, except for the bottleneck, where no pooling is applied. Each decoder stage uses bilinear up-sampling to double the spatial resolution, followed by a $\:3\times\:3$ convolution, then concatenation with the corresponding attention-gated encoder features from the same level (via skip connections), and finally two residual-SE blocks for refinement.

Table 2 provides a detailed layer-wise configuration of the proposed W-AGRU-Net, including spatial dimensions, number of channels, residual-SE blocks, and attention gate connections.

**Table 2:** Detailed architectural configuration of the proposed W-AGRU-Net.

Stage	Input size	Channels (in/out)	#Res-SE blocks	Down-sampling (MaxPool)	Attention gate (AG)
Encoder 1	256 × 256	32	2	Yes (2 × 2)	No
Encoder 2	128 × 128	64	2	Yes	Yes (from Decoder 3)
Encoder 3	64 × 64	128	2	Yes	Yes (from Decoder 2)
Encoder 4	32 × 32	256	2	Yes (to 16 × 16)	Yes (from Decoder 1)
Bottleneck	16 × 16	512	2	No	No
Decoder 1 (up)	16 × 16 → 32 × 32	512 → 256	2	No (bilinear up)	No
Decoder 2	32 × 32 → 64 × 64	256 → 128	2	No	Yes (from Encoder 3)
Decoder 3	64 × 64 → 128 × 128	128 → 64	2	No	Yes (from Encoder 2)
Decoder 4	128 × 128 → 256 × 256	64 → 32	2	No	Yes (from Encoder 1)
Output	256 × 256	1 (sigmoid)	–	–	–

All convolutional layers (except the final $\:1\times\:1$ output layer) Use a kernel size of $\:3\times\:3$, padding = * 1*, and He normal initialization. Batch normalization is applied after every convolution before the activation function. The final output layer is a $\:1\times\:1$ convolution followed by a sigmoid activation to produce a pixel-wise probability map.

### Hybrid loss function

For tumor versus background segmentation, we developed a hybrid loss function, integrating Binary Cross Entropy (BCE) and Dice loss. This accounts for both pixel-wise classification and spatial overlap, especially concerning imbalanced medical imaging.

Let $\:N=256\times\:256$ be the total number of pixels in an image. For each pixel * i*, let $\:{y}_{i}\in\:\left\{\mathrm{0,1}\right\}$ be the ground-truth label and $\:{\widehat{y}}_{i}\in\:\left[\mathrm{0,1}\right]$ the predicted probability from the sigmoid output.

**Binary Cross-Entropy (BCE) loss**:

15 $$\:\begin{array}{cccc}&\:{\mathcal{L}}_{\mathrm{BCE}}=-\frac{1}{N}\sum\limits_{i=1}^{N}\left[{y}_{i}\mathrm{l}\mathrm{o}\mathrm{g}\left({\widehat{y}}_{i}\right)+(1-{y}_{i})\mathrm{l}\mathrm{o}\mathrm{g}(1-{\widehat{y}}_{i})\right].&\:&\:\end{array}$$

**Dice loss** (based on the Sørensen–Dice coefficient):

16 $$\:\begin{array}{cccc}&\:{\mathcal{L}}_{\mathrm{Dice}}=1-\frac{2\sum\:_{i=1}^{N}{y}_{i}{\widehat{y}}_{i}+\epsilon}{\sum\:_{i=1}^{N}{y}_{i}+\sum\:_{i=1}^{N}{\widehat{y}}_{i}+\epsilon}&\:&\:\end{array}$$

where $\epsilon={10}^{-6}$ is a smoothing constant to prevent division by zero.

**Hybrid loss**:

17 $$\:\begin{array}{cccc}&\:{\mathcal{L}}_{\mathrm{Hybrid}}=\alpha\:{\mathcal{L}}_{\mathrm{BCE}}+\beta\:{\mathcal{L}}_{\mathrm{Dice}},&\:&\:\end{array}$$

with $\:\alpha\:=0.6$ and $\:\beta\:=0.4$ determined empirically through grid search. This weighting balances the pixel-wise classification accuracy (BCE) and the structural similarity (Dice).

**Final output activation** (sigmoid) for a given input * X*:

18 $$\:\begin{array}{cccc}&\:P\left(X\right)=\frac{1}{1+{e}^{-(WX+b)}},&\:&\:\end{array}$$

where * W* and * b* are the weights and bias of the final $\:1\times\:1$ convolutional layer, and $\:P\left(X\right)\in\:[\mathrm{0,1}{]}^{256\times\:256}$ is the probability map for tumor presence. Each value in $\:P\left(X\right)$ represents the probability that the corresponding pixel belongs to the tumor class (foreground), while values near * 0* indicate background. This formulation explicitly defines a pixel-wise binary classification task, which aligns directly with the ground-truth masks $\:{\left\{\mathrm{0,1}\right\}}^{256\times\:256\times\:1}$ and with the hybrid loss $\:{\mathcal{L}}_{\mathrm{Hy}\mathrm{brid}}$ that combines BCE (for pixel-wise accuracy) and Dice (for structural overlap).

For training, the model applies the Adam optimizer for a batch size of 16 (for TCIA LGG) or 4 (for Figshare) and for a learning rate of 10^− 4^. Early stopping with a patience of 15 epochs is preserved during training to account for overfitting.

Consequently, W-AGRU-Net integrates attention mechanisms, enhanced residual learning, and channel recalibration to improve tumor boundary delineation while maintaining robustness to inter- and intra-tumor variations in size, shape, and intensity. This design also improves the detection of small pathological regions.

## Simulation and results

This section provides a complete analysis of the W-AGRU-Net framework, evaluated on two benchmark datasets. The experimental setup, data pre-processing pipeline, implementation details, and detailed performance analysis are presented in this section.

### Datasets and data splitting

#### Dataset 1: Figshare CE-MRI dataset

The Figshare dataset consists of 3,064 T1-weighted contrast-enhanced MRI scans from 233 patients with three different types of brain tumors: pituitary tumors, gliomas, and meningiomas. The dataset includes comprehensive annotations consisting of a patient identifier, precise tumor boundaries, diagnostic labels, and an accompanying tumor mask that clearly delineates pathological areas. This dataset is ideal for examining the robustness of segmentation algorithms in the evaluation of different pathological presentations due to its mixture of tumor types and sizes.

#### Dataset 2: TCIA-LGG segmentation dataset

**Fig. 4:**
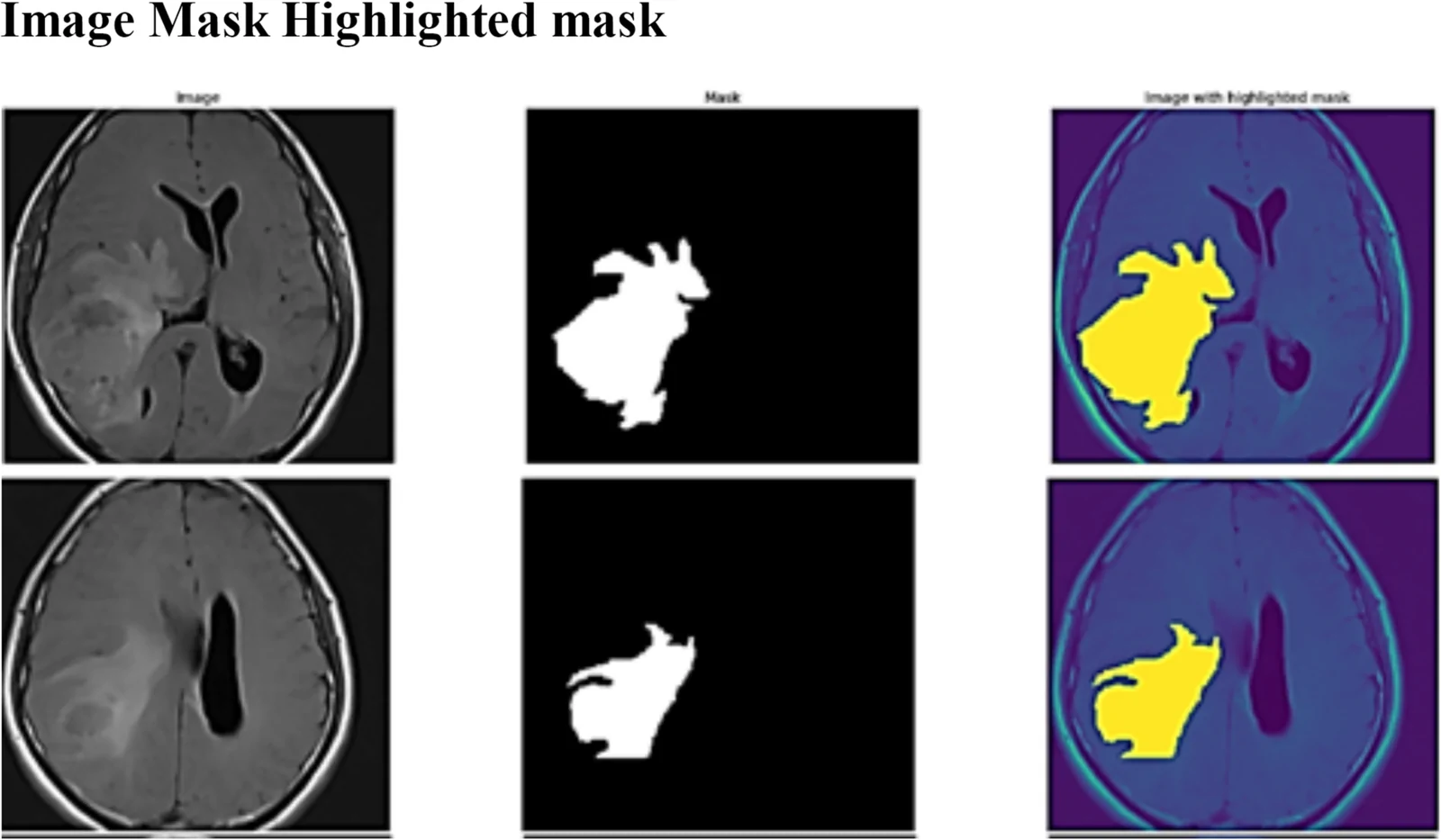
LGG -TCIA random sample images with their equivalent masks.

The TCIA-LGG dataset is a consortium dataset of 120 patients with lower-grade gliomas (LGGs) from The Cancer Genome Atlas (TCGA)^36^ and The Cancer Imaging Archive (TCIA)^37^. The dataset includes multi-parametric imaging data with FLAIR, pre- and post-contrast T1-weighted images, along with genomic profiling. Within the total cohort of patients, 110 were systematically classified into 22 molecular clusters based on IDH mutation status and DNA methylation patterns to provide a richly informative dataset to assess segmentation performance in clinically relevant settings. Figure 4 illustrates example cases from the cohort, which demonstrate the breadth of tumor appearance and locations.

#### Data set partitioning

**Fig. 5:**
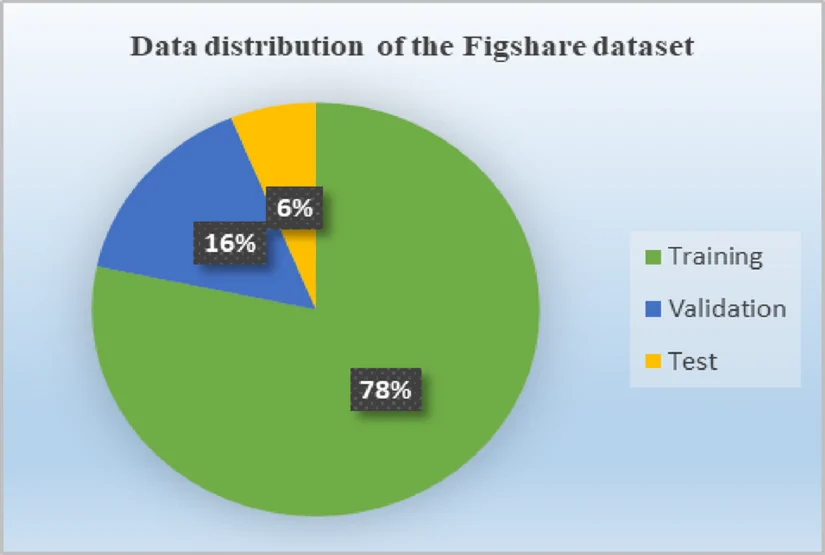
The division of the dataset on brain tumors for testing, validation, and training.

Both datasets were partitioned using a random splitting procedure in proportions of 78% for training, 16% for validation, and 6% for testing. The dataset split was performed at the slice level due to the absence of explicit patient-wise grouping in the implemented pipeline. All images were randomly shuffled before partitioning into training, validation, and test sets. This partitioning strategy was adopted to maximize the utilization of limited annotated data while ensuring consistent evaluation across all experiments. The training set was used for model optimization, the validation set for hyperparameter tuning, and the test set for final performance evaluation. The same splitting strategy was applied consistently across all compared models to ensure fair comparison. The division of the dataset is illustrated in Fig. 5.

### Data preprocessing pipeline

**The proposed preprocessing pipeline addresses key challenges in brain MRI analysis**, including intensity inhomogeneity, contrast variation, and noise artifacts. All of these factors can influence joint probability estimation, which will affect segmentation performance.

#### Noise removal and edge preservation

The bilateral filtering technique was chosen to reduce noise while sustaining the important edge structures. A bilateral filter is considered a non-linear smoothing approach that utilizes statistical combinations on a domain (filter size) and range (pixel intensity difference), mathematically expressed as:

18 $$\:{\boldsymbol{B}\boldsymbol{F}\left[\boldsymbol{I}\right]}_{\boldsymbol{p}=\frac{1}{{\boldsymbol{W}}_{\boldsymbol{p}}}}\sum\:_{\boldsymbol{q}\in\:\boldsymbol{S}}{\boldsymbol{G}}_{{\boldsymbol{\sigma\:}}_{\boldsymbol{S}}}\left(\left|\left|\boldsymbol{p}-\boldsymbol{q}\right|\right|\right){\boldsymbol{G}}_{{\boldsymbol{\sigma\:}}_{\boldsymbol{r}}}\left(\right|{\boldsymbol{I}}_{\boldsymbol{p}}-{\boldsymbol{I}}_{\boldsymbol{q}}\left|\right){\boldsymbol{I}}_{\boldsymbol{q}}$$

where:

- $\:{G}_{{\sigma\:}_{S}}$ represents the spatial Gaussian kernel controlling the influence of neighboring pixels based on spatial proximity.
- $\:{G}_{{\sigma\:}_{r}}$ denotes the range Gaussian kernel that preserves edges by considering intensity differences.
- $\:{W}_{p}$ is the normalization factor ensuring filter weights sum to unity.
- * S* defines the spatial neighborhood around the pixel * p*.

#### Standardizing spatial dimensions and normalizing intensity

**Fig. 6:**
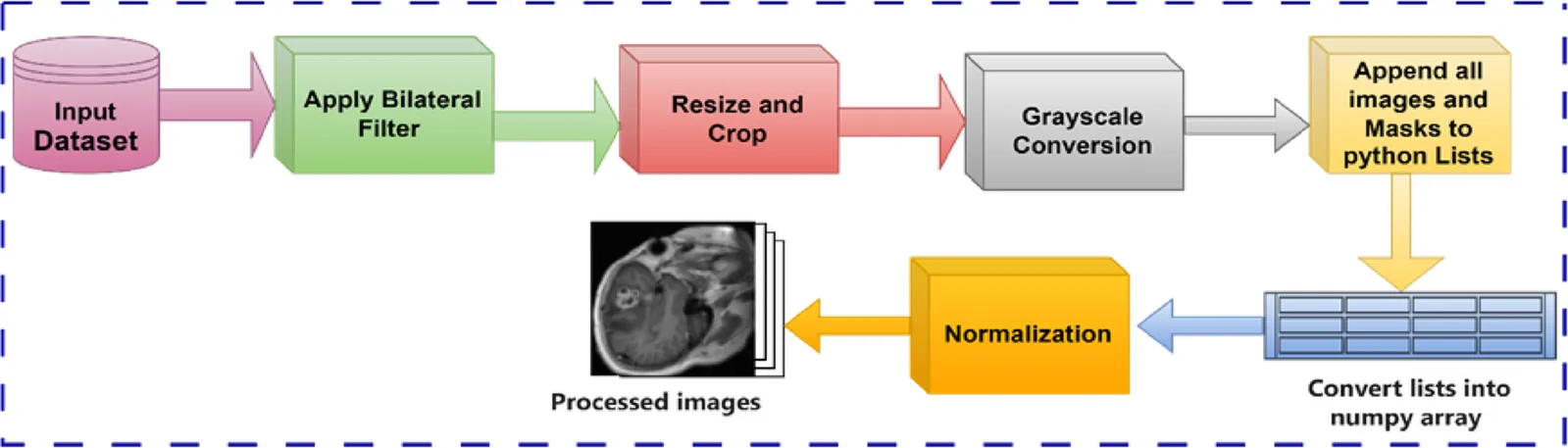
Schematic steps of preprocessing for the proposed system.

The filtered images underwent spatial standardization by resizing and cropping to ensure that all samples had the same dimensions. Using the same spatially standardized images, the intensity normalization procedure was implemented to normalize dynamic range across different MRI scans and to account for variations in contrast and brightness. The normalization rule applies:

19 $$\:{\boldsymbol{I}}_{\boldsymbol{n}\boldsymbol{o}\boldsymbol{r}\boldsymbol{m}}=\frac{\boldsymbol{I}-{\boldsymbol{I}}_{\boldsymbol{m}\boldsymbol{i}\boldsymbol{n}}}{{\boldsymbol{I}}_{\boldsymbol{m}\boldsymbol{a}\boldsymbol{x}}-{\boldsymbol{I}}_{\boldsymbol{m}\boldsymbol{i}\boldsymbol{n}}}$$

where $\:{I}_{min}$ and $\:{I}_{max}$ are the minimum and maximum pixel intensity values within each image volume, which normalize all intensity values into the [0, 1] range. Hence, intensity normalization improved training stability and accelerated the convergence of the model. All MRI slices were resized to a fixed spatial resolution of 256 × 256 pixels to ensure uniform input representation across the dataset. The normalized images were then used as input to the proposed model. The input to the network consists of single-channel (grayscale) MRI images, as both the Figshare and TCIA-LGG datasets provide single-modality scans. The entire preprocessing workflow is shown in Fig. 6.

### Metrics for evaluation

The segmentation task is formulated as a binary classification problem, where each pixel is categorized as either tumor (foreground) or non-tumor (background). Accordingly, the evaluation metrics are computed based on pixel-wise classification outcomes. The study employs standard evaluation metrics for brain tumor segmentation, including DSC, Jaccard, Accuracy, Precision, Sensitivity, and Loss. Jaccard, also known as the IoU index, quantifies overlap between regions. Correctly segmented and incorrectly segmented brain tumors are represented by TP, FP, and FN. Precision (Pre) measures the proportion of accurate predictions, while sensitivity (recall score) is determined by dividing the total number of positive samples by the actual positives. Accuracy (Acc) measures the overall performance of pixel-level segmentation.

20 $$\:Jaccard=\frac{Tp}{Tp+Fp+FN}$$

21 $$\:\:\:\:\:\:\:\:DSC=\frac{Tp}{2Tp+Fp+FN}$$

22 $$\:\:\:\:\:\:Pre=\frac{Tp}{Tp+Fp}$$

23 $$\:\mathrm{S}\mathrm{e}\mathrm{n}\mathrm{s}\mathrm{i}\mathrm{t}\mathrm{i}\mathrm{v}\mathrm{i}\mathrm{t}\mathrm{y}\:=\frac{Tp}{Tp+FN}$$

24 $$\:Acc=\frac{\boldsymbol{T}\boldsymbol{p}+\boldsymbol{T}\boldsymbol{N}}{\boldsymbol{T}\boldsymbol{p}+\boldsymbol{T}\boldsymbol{N}+\boldsymbol{F}\boldsymbol{p}+\boldsymbol{F}\boldsymbol{N}}$$

### Implementation details and experimental setup

The proposed W-AGRU-Net framework was implemented using the Keras DL library inside the TensorFlow 2.8 environment. All experiments were conducted on a high-performance computing system equipped with NVIDIA Tesla V100 GPUs and 32GB of memory. The model was trained for 100 epochs using the Adam optimizer with an initial learning rate of 0.0001, selected through preliminary experiments for stable convergence. Careful hyperparameter tuning was performed to determine the optimum configuration for brain tumor segmentation, combining systematic grid search and manual tuning. The training process included early stopping with a patience of 15 epochs to prevent overfitting and reduce computational resources. A summary of the hyperparameter configuration is given in Table 3.

**Table 3:** Hyperparameter Configuration for W-AGRU-Net Model.

Parameter	Value	Description
Framework	Keras with TensorFlow 2.8	Deep learning environment
Optimizer	Adam	Adaptive moment estimation
Learning Rate	0.0001	Initial learning rate
Batch Size	16 (TCIA-LGG), 4 (Figshare)	Dataset-specific batch sizes
Epochs	100	Maximum training epochs
Early Stopping	15 epochs	Patience for convergence detection
Activation Function	ReLU	Encoder/decoder non-linearity
Final Activation	Sigmoid	Output layer activation
Weight Initialization	He Normal	Convolutional layer initialization
Regularization	L2 (λ = 0.0001)	Weight decay parameter

### Hybrid loss function formulation

To address various difficulties in medical image segmentation, such as imbalanced classification problems and ambiguity regarding boundaries, we devised a hybrid loss function that combines Binary Cross-Entropy (BCE)and Dice loss components. The Binary Cross-Entropy loss function^38^ quantifies the dissimilarity on a pixel-wise basis between predicted segmentation masks relative to the corresponding annotations, as defined in the following equation:

25 $$\:{\:\:\:\:\:\:\:\:\:\:\:\:\:\:\:\:\:\:\:\:\:\:\:\:\:\:\boldsymbol{L}}_{\boldsymbol{B}\boldsymbol{C}\boldsymbol{E}}=-\frac{1}{\boldsymbol{n}}\sum\:_{\boldsymbol{i}=1}^{\boldsymbol{n}}\{{\boldsymbol{y}}_{\boldsymbol{i}}\boldsymbol{log}{\boldsymbol{f}}_{\boldsymbol{i}}+(1-{\boldsymbol{y}}_{\boldsymbol{i}})\:\boldsymbol{log}{(1-\boldsymbol{f}}_{\boldsymbol{i}}\left)\right\}$$

where $\:{y}_{i}$ represents the ground truth label and $\:{\widehat{y}}_{i}$ denotes the predicted probability for the pixel * i*, with * N* indicating the total number of pixels.

In addition to the BCE loss, the Dice loss function increases boundary detection fidelity and ensures segmentation fidelity by estimating spatial overlap between predicted and target regions:

26 $$\:{\mathcal{L}}_{Dice}=1-\frac{2\sum\:_{i=1}^{N}{y}_{i}{\widehat{y}}_{i}+\epsilon}{\sum\:_{i=1}^{N}{y}_{i}+\sum\:_{i=1}^{N}{\widehat{y}}_{i}+\epsilon}$$

where $\epsilon=1\times\:{10}^{-6}$ is a smoothing constant added to prevent numerical instability during training.

The final hybrid loss function integrates both components through weighted summation:

27 $$\:{\mathcal{L}}_{Hybrid}=\alpha\:\cdot\:{\mathcal{L}}_{BCE}+\beta\:\cdot\:{\mathcal{L}}_{Dice}\:\:\:\:\:\:\:\:\:\:\:\:\:\:\:\:\:\:\:\:\:\:\:\:\:\:\:\:\:\:\:\:\:\:$$

with α = 0.6 α = 0.6 and β = 0.4 β = 0.4 determined through experimentation to balance the contributions of the two losses. Specifically, the BCE loss provides the model with a robust pixel-wise classification procedure, while the Dice loss enforces a sense of structural similarity while addressing class imbalance, especially relevant in brain tumor segmentation, leading to improved convergence stability and segmentation performance through the dual loss.

### Experimental results and discussion

To evaluate the contribution of each architectural component, we implemented a series of baseline and intermediate model variants. Specifically, U-Net^4^, U-Net++^39^, and AGRU-Net^5^ were re-implemented from the literature under identical experimental conditions, while AGU-Net, W-U-Net, and the proposed W-AGRU-Net were developed as intermediate architectural variants for the ablation study. All models were trained using the same data split (78%/16%/6%, Sect. 4.1.3), the same preprocessing pipeline (Sect. 4.2), and the same hardware (NVIDIA Tesla V100 GPU). None of the reported results were taken directly from previous publications; instead, all experiments were conducted by the authors to ensure a fair and unbiased comparison.

To fairly evaluate the contribution of each architectural component, we performed a series of ablation experiments based on progressively extending the original U-Net architecture. Starting from U-Net++, we successively introduced the dual-branch architecture (W-U-Net), attention gates (AGU-Net), attention-guided residual learning (AGRU-Net), and finally the complete proposed W-AGRU-Net. Within this progressive design, Pyramid-Shaped Dilated Convolution Schemes (PDCS) were incorporated into the dual-branch architecture to enhance multi-scale contextual representation, while Squeeze-and-Excitation (SE) blocks were integrated into the residual units to improve channel-wise feature recalibration (Table 4).

**Table 4:** Ablation study demonstrating the contribution of different model components: Dice and Jaccard scores for each variant on Figshare and LGG-TCIA datasets.

Model	Figshare dataset		TCIA-LGG dataset	
	Dice (Mean ± SD)	Jaccard (%) (Mean ± SD)	Dice (%)	Jaccard (%)
U-Net	92.31 ± 0.27	84.29 ± 2.43	91.78	84.75
U-Net++	91.73 ± 0.36	85.08 ± 0.87	92.10	85.27
AGU-Net	93.47 ± 0.17	87.29 ± 0.45	92.67	86.18
W-U-Net	93.59 ± 0.31	86.08 ± 0.25	92.57	85.95
W-AGU-Net	97.43 ± 0.25	92.62 ± 0.70	93.05	87.92
AGRU-Net	96.37 ± 0.22	95.78 ± 0.30	93.43	88.79
**W-AGRU-Net**	**97.87 ± 0.18**	**97.44 ± 0.05**	**94.00**	**89.29**

The ablation study isolates the contribution of each architectural component incrementally: (i) dual-branch cascade (W-U-Net), (ii) attention gates (AGU-Net), (iii) residual-SE units (AGRU-Net), and (iv) pyramid-shaped dilated convolutions (PDCS) within the dual-branch cascade (W-AGRU-Net). This stepwise design allows clear attribution of performance gains to each component.

#### Statistical significance analysis

To assess the statistical significance of the observed performance differences, a one-way ANOVA was conducted on the Dice and Jaccard scores obtained from three independent runs on the Figshare dataset using different random seeds while maintaining the same data split and experimental settings. A significance level of *p* < 0.05 was adopted. The analysis revealed statistically significant differences among the evaluated models for both Dice (F = 332.02, *p* < 0.001) and Jaccard (F = 98.14, *p* < 0.001). These findings indicate that the observed performance differences among the evaluated models were statistically significant under the adopted experimental protocol.

#### Full performance analysis

The quantitative analysis demonstrates the effectiveness of the proposed architectural design for brain tumor segmentation. The W-AGRU- Net achieves state-of-the-art performance on the Figshare dataset, attaining a Dice score of 97.87% and a Jaccard score of 97.44% (averaged over 3 runs), and representing a 5.56% improvement in Dice over the standard U-Net (92.31%).

The systematic exploration of architectural evolution demonstrates distinct patterns of performance enhancement, as follows:

- Dual-branch cascade (W-U-Net) yields up to 0.62% Dice improvement over basic U-Net.
- Attention gates improve feature discrimination, particularly around complex tumor boundaries.
- Residual connections with SE blocks (AGRU-Net) produce substantially stronger performance, achieving 96.37% Dice on Figshare.
- The complete W-AGRU-Net, combining all components (dual-branch cascade, attention gates, residual-SE units, and PDCS modules), delivers the highest overall performance with **97.87%** Dice on Figshare.

#### Cross-dataset validation and robustness

The consistent advantage of W-AGRU-Net demonstrates consistent performance across both datasets. On the TCIA-LGG dataset, our proposed model achieved a Dice coefficient of **94.0%** and a Jaccard index of **89.29%**, outperforming all baseline models in terms of Dice. Notably, while simpler baselines such as U-Net and U-Net + + showed lower Dice scores (91.78% and 92.10%, respectively), their corresponding Jaccard values (84.75% and 85.27%) remained consistently lower than those of our proposed model. This confirms that the performance gains of W-AGRU-Net are both statistically and practically significant. Our model showed particularly encouraging stability across tumor types and imaging protocols, suggesting its potential for future clinical applications following further patient-wise and external validation.

**Fig. 7:**
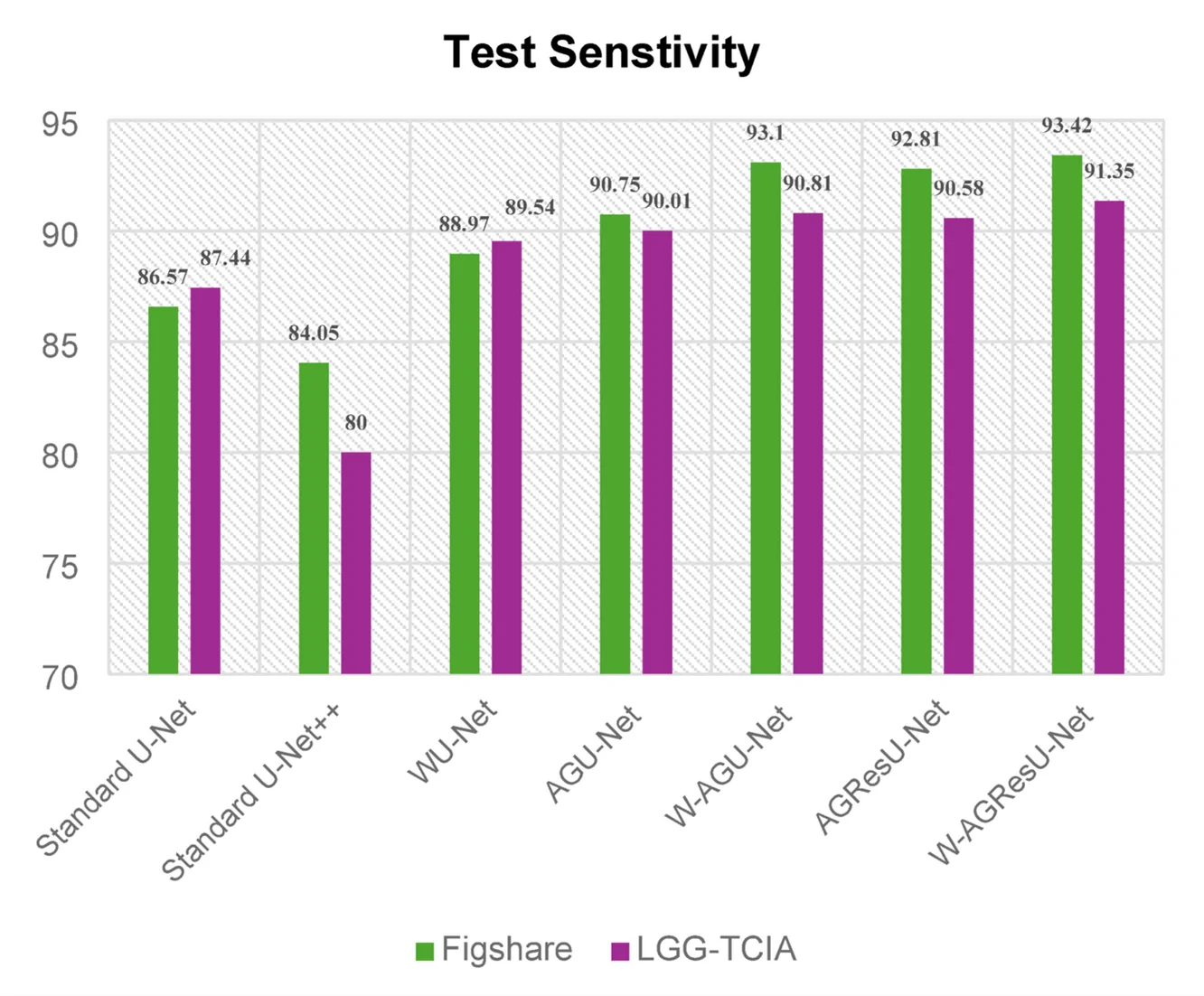
Performance Segmentation of the proposed model regarding sensitivity.

As shown in Fig. 7, our model achieved a sensitivity of **93.42%** on the Figshare dataset and **91.35%** on the TCIA-LGG dataset. These represent improvements of 6.85% and 3.91%, respectively, compared to the standard U-Net, demonstrating the model’s enhanced ability to detect true positive tumor regions.

**Fig. 8:**
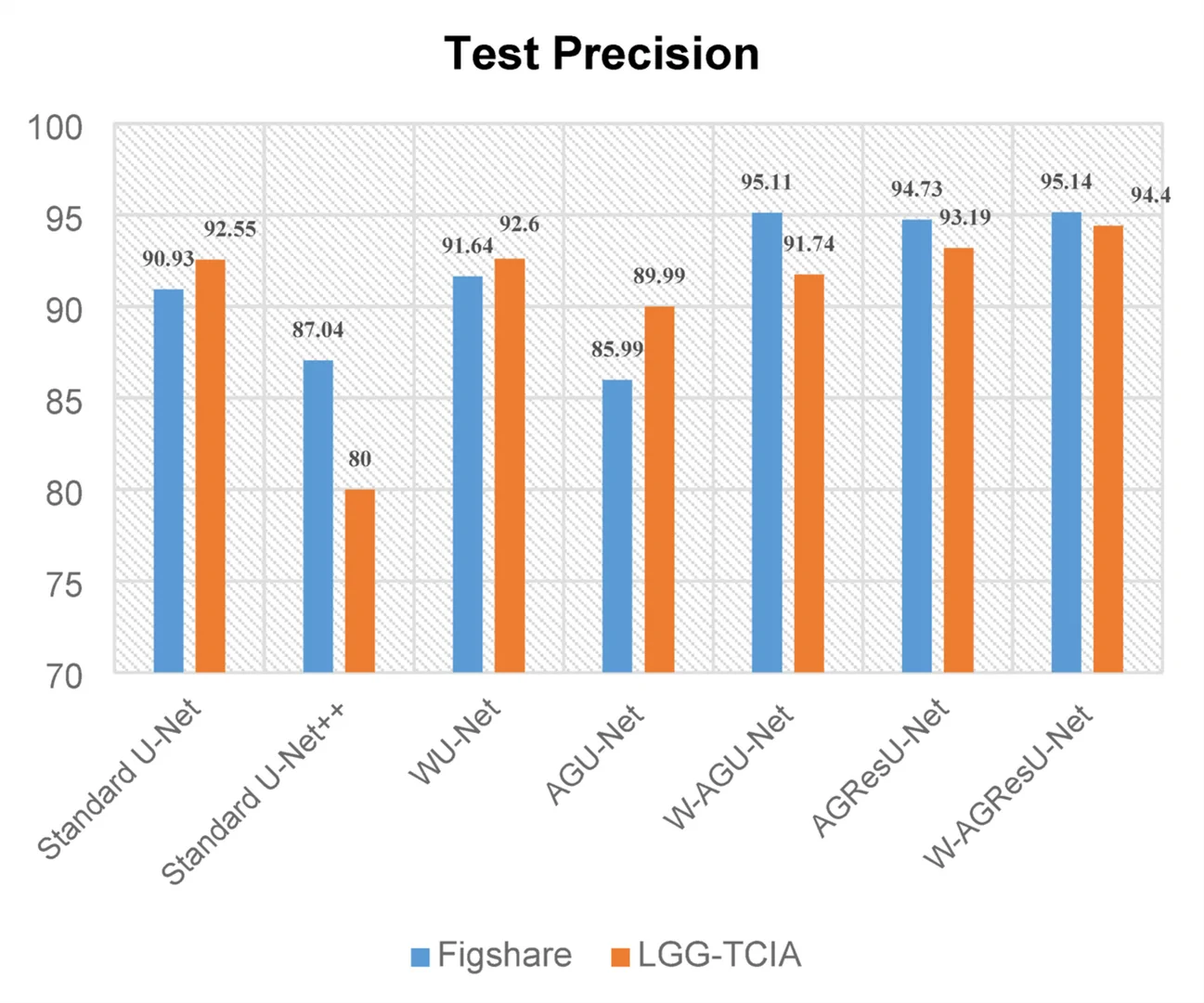
Performance Segmentation of the proposed model regarding precision.

Similarly, Fig. 8 shows improved precision metrics over baseline models. W-AGRU-Net outperformed the individual AGRU-Net by 0.61% in precision on Figshare and by 0.77% on TCIA-LGG, confirming the model’s ability to reduce false positive predictions.

**Fig. 9:**
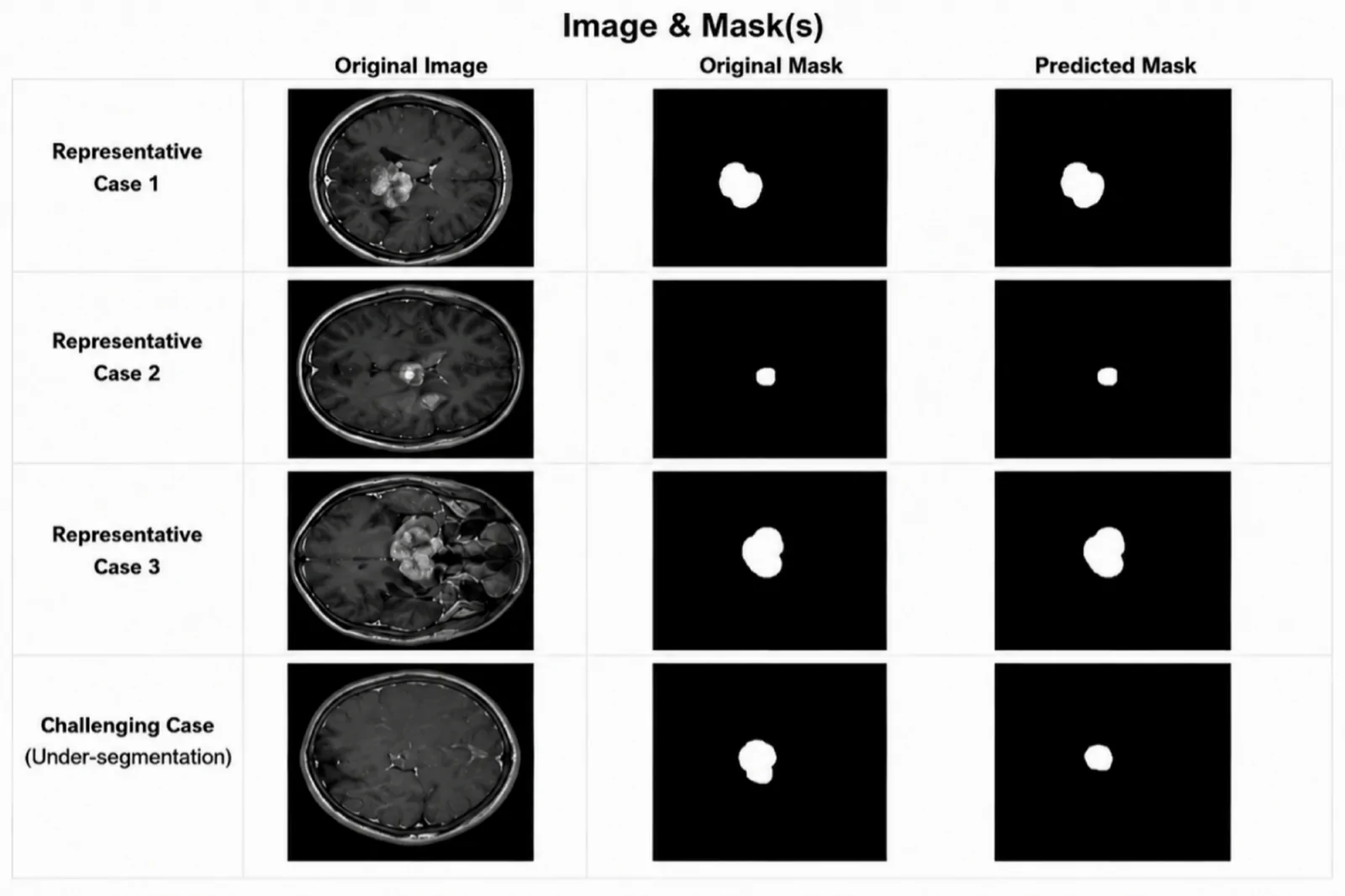
Representative qualitative segmentation results of the proposed W-AGRU-Net on the Figshare dataset. The first three rows show representative cases with close agreement between the predicted and ground-truth masks, whereas the last row presents a challenging case with slight under-segmentation.

To further investigate the behavior of the proposed W-AGRU-Net beyond the quantitative evaluation, a qualitative analysis was conducted on representative test samples. As shown in Fig. 9, the first three examples demonstrate close agreement between the predicted masks and the corresponding ground-truth annotations, indicating accurate tumor localization and boundary delineation. In contrast, the last example represents a challenging case in which the model produced slight under-segmentation, missing a small portion of the tumor region. Such errors were mainly observed in slices with irregular tumor boundaries or relatively low contrast between the tumor and surrounding tissues. Nevertheless, these cases were infrequent and had a limited impact on the overall segmentation performance, as reflected by the consistently high Dice and Jaccard scores. These observations are consistent with previous studies, which have reported that small lesions, indistinct tumor boundaries, and low-contrast regions remain among the most challenging scenarios for CNN-based brain tumor segmentation. The relatively limited number of failure cases observed in our experiments suggests that the proposed W-AGRU-Net provides improved robustness compared with the evaluated baseline models.

#### Training behavior and convergence assessment

**Fig. 10:**
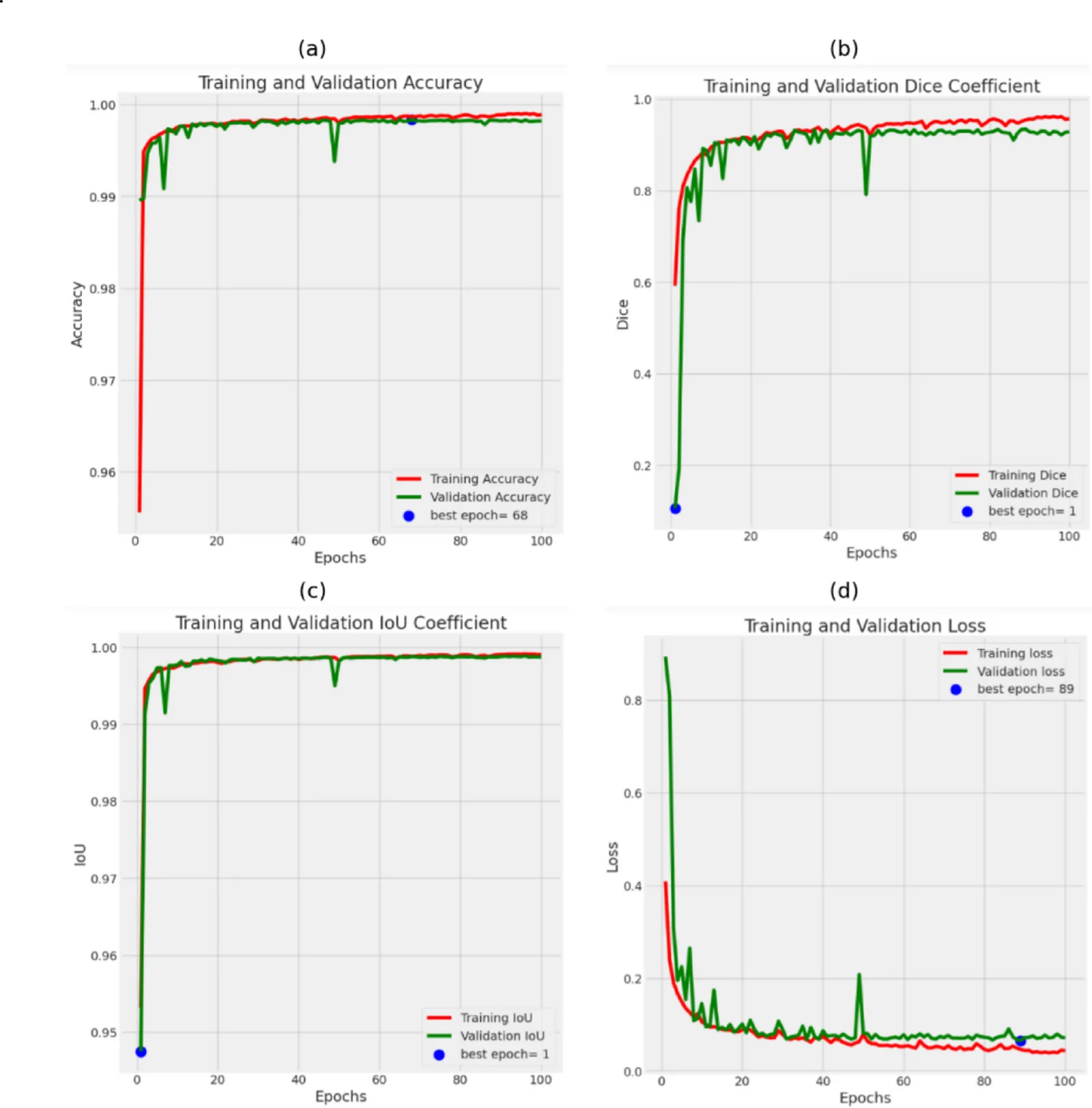
Training and validation performance on the TCIA dataset: (**a**) Accuracy, (**b**) Dice coefficient, (**c**) IoU, and (**d**) Loss.

**Fig. 11:**
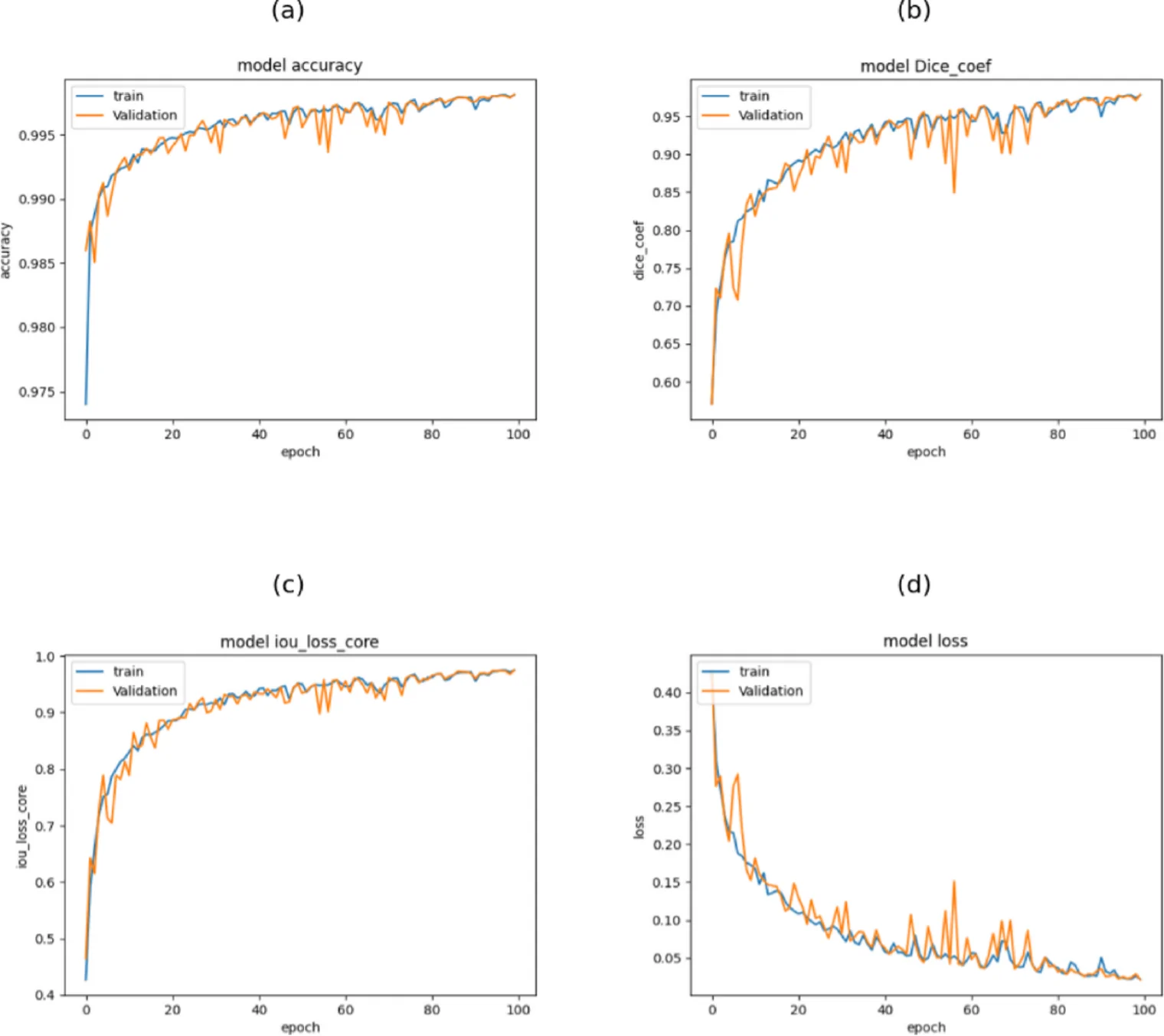
Training and validation performance on the Figshare dataset: (**a**) Accuracy, (**b**) Dice coefficient, (**c**) IoU, and (**d**) Loss.

The training behavior, demonstrated through visualizations in Fig. 10 (TCIA) and Fig. 11 (Figshare), exhibited favorable convergence characteristics with few signs of overfitting. The pairs of training and validation curves were consistent for all metrics (accuracy, Dice coefficient, IoU, and loss), which indicates a consistent learning behavior of the model and an impressive generalization ability.

#### Qualitative results and visual evidence

**Fig. 12:**
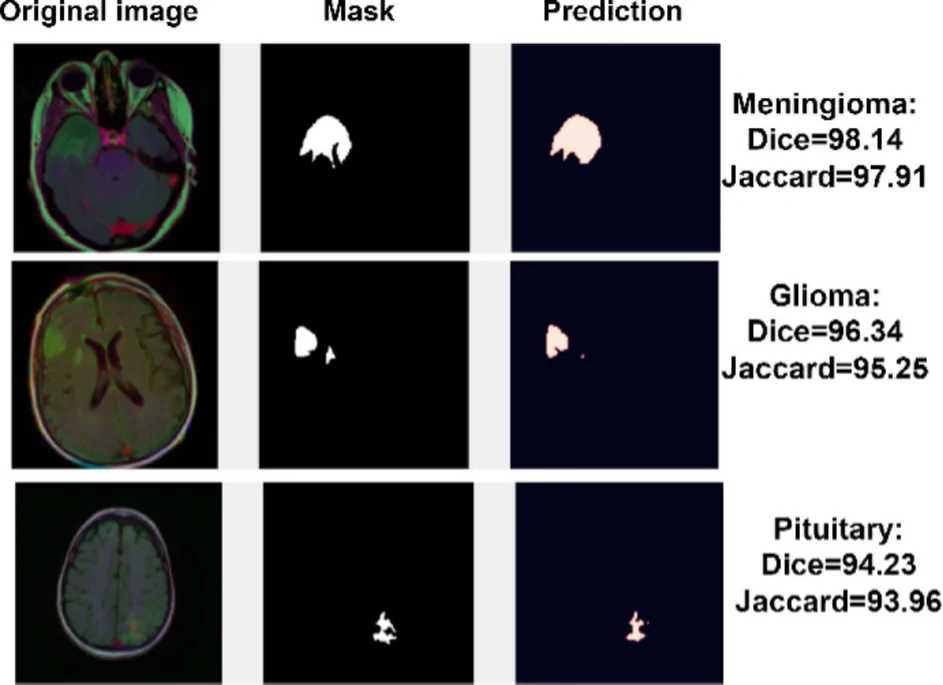
Segmentation output of the W-AG-Res-U-Net vs. ground truth.

Figure 12 shows visual evidence of the segmentation quality obtained through leveraging W-AGRU-Net by presenting effective tumor boundary demarcation and exact spatial localization for different tumor morphologies. The qualitative results confirm the quantitative results and illustrate how the model handles different tumor sizes, shapes, and intensity patterns while maintaining anatomical correctness relative to the ground truth.

The results achieved by W-AGRU-Net outperformed the other models due to the collaborative integration of each novel component: the two-stream architecture that endows multi-scale feature learning, the attention mechanisms that select salient features, the residual connection and the SE blocks that enable the better gradient flow and feature recalibration, and the hybrid loss function that alleviates the class imbalance problem that is inherent to medical image segmentation tasks.

### Computational complexity and efficiency analysis

We performed an end-to-end analysis of the computational and efficiency metrics to evaluate the practical significance of the proposed architecture. Table 5 provides a comprehensive evaluation of model complexity, training efficiency, and inference time for each variant included in this ablation study.

For each dataset, all inference-time measurements were performed under identical experimental conditions, including the same hardware (NVIDIA Tesla V100 GPU), the same input resolution (256 × 256), and the same evaluation batch size, ensuring a fair comparison among all models.

**Table 5:** Computational Complexity and Efficiency Comparison.

Model	Total Parameters	Total Training Time (s)	Average Time/Epoch (s)	Inference Time/Step (s)
U-Net	7,771,297	3,510.6	35.1	0.80
W-U-Net	13,346,305	7,100.19	71.0	0.77
AGU-Net	11,021,381	3,825.13	38.25	0.55
AGRU-Net	11,295,913	5,429.15	54.29	0.33
W-AGU-Net	19,606,697	12,221.90	122.22	0.49
W-AGRU-Net (Proposed)	**20**,**955**,**561**	**11**,**319.20**	**113.19**	**0.261**

While the proposed W-AGRU-Net has 20.96 million parameters (more than U-Net’s 7.77 M), its inference speed of 0.261 s/step is 67.4% faster than U-Net (0.80 s/step). This demonstrates that parameter count alone does not determine inference latency; architectural design and hardware-efficient operations (depthwise separable convolutions, parallel streams, and lightweight attention gates) play a critical role. We observe a good balance of model capacity and computational cost in the new W-AGRU-Net design. The model was configured with 20.96 million parameters, therefore a more complex model than U-Net (7.77 M) and even the middle-of-the-road models, yet provided the most efficient ratio of training speed to time, with a 11,319.2 s total training time (113.19 s per epoch). There was a 7.4% gain in training speed efficiency, even with more parameters than W-AGU-Net, using standardized training with 122.22 s per epoch.

Notably, the W-AGRU-Net demonstrated exceptional inference speed with a maximum average processing time of 0.261 s per step, significantly outperforming all baseline models as follows:

- 67.4% faster compared to standard U-Net (0.80 s/step).
- 66.1% faster compared to W-U-Net (0.77 s/step).
- 52.5% faster compared to AGU-Net (0.55 s/step).
- 20.9% faster compared to AGRU-Net (0.33 s/step).
- 46.7% faster compared to W-AGU-Net (0.49 s/step).

This level of inference speed, in tandem with state-of-the-art segmentation performance, positions W-AGRU-Net as a promising candidate for future real-time clinical applications, subject to further validation.

### Comparative analysis with state-of-the-art methods

Tables 6 and 7 provide extensive comparative analysis against recent state-of-the-art methods on the TCIA-LGG and Figshare datasets, respectively.

**Table 6:** Performance Comparison on TCIA-LGG Dataset.

Reference	Method	Dice (%)	Jaccard (%)	Accuracy (%)
Bentaher et al.^40^	Attention + Residual	92	86	-
Behzadpour et al.^41^	Transfer Learning + Attention	79.7	-	-
Hossain et al.^42^	DeeplabV3+	73.56	60.06	-
Buchade et al.^43^	3D CNN + U-Net	92	-	98.5
Ghosh et al.^44^	U-Net + VGG16	92	-	99.75
W-AGRU-Net (Proposed)	**Dual Attention Residual UNet**	**94.0**	**89.29**	**99.80**

**Table 7:** Comparison of the proposed W-AGRU-Net with recent state-of-the-art methods evaluated on the Figshare brain tumor MRI dataset.

Reference	Method	Dice (%)	Jaccard (%)	Accuracy (%)
**Sobhaninia et al.**^45^**(2020)**	Cascaded CNN	80.00	90.70	-
Masood et al.^34^(2021)	Traditional Mask R-CNN	95.00	95.00	95.10
Masood et al.^34^(2021)	Mask R-CNN (ResNet-50)	95.50	95.10	95.90
Shanaka et al.^46^(2021)	R-CNN + Chan-Vese	92.00	-	94.57
Francisco et al.^47^(2021)	CNN	82.80	-	-
Li et al.^30^(2023)	Multitask + MMRAN	80.03	84.38	89.97
S. Rajendran et al.^48^(2023)	3CNN + U-Net	-	81.96	98.54
S. A. Zargari et al.^49^(2023)	O2U-Net	80.83	-	99.34
S. Saifullah et al.^50^(2024)	DeepLabV3+ + ResNet18	91.23	93.40	97.48
S. Saifullah et al.^13^(2024)	U-Net + PSO	94.14	89.02	-
S. Saifullah et al.^10^(2024)	DeepLabV3 + + ResNet50	96.90	94.04	99.93
S. Saifullah et al.^51^(2025)	ResNet50 + U-Net	95.53	91.15	-
Preetha et al.^31^(2025)	Multi-scale Attention + EfficientNetB4	93.39	87.95	99.79
Chen et al.^32^(2026)	YOLO-LS	91.00	-	-
Abbas et al.^29^(2026)	Dual-Encoder U-Net	95.27	86.89	99.00
**W-AGRU-Net (Proposed)**	**W-AGRU-Net**	**97.87**	**97.44**	**99.80**

The comparative study showed that each architectural component contributed to the overall performance improvement. Specifically, attention gates enhanced tumor boundary localization, residual SE units improved feature recalibration, and the pyramid-shaped dilated convolution schemes strengthened multi-scale contextual feature extraction within the dual-stream architecture. As shown in Tables 6 and 7, the proposed W-AGRU-Net achieved the best segmentation performance on the Figshare dataset and remained highly competitive on the TCIA-LGG dataset. These findings suggest that integrating complementary architectural components within a unified dual-stream framework is more effective than applying attention mechanisms, residual learning, or multi-scale feature extraction independently. The observed improvements are consistent with recent studies highlighting the importance of attention mechanisms and multi-scale contextual learning for medical image segmentation, while also indicating that combining these complementary strategies within a unified architecture can provide additional performance gains.

### Limitations and future directions

The current research acknowledges several limitations that inform possibilities for future research. First, the evaluation was conducted on two publicly available datasets. The TCIA-LGG dataset is primarily comprised of FLAIR images only, and the Figshare dataset only contained T1-weighted images. Both these datasets are relatively small in comparison to larger datasets and are not multi-modal MRI datasets that would typically be available in clinical settings. Current benchmark datasets, such as BraTS, provide multi-modal inputs, such as T1, T1Gd, T2, and FLAIR sequences, to provide more anatomical and pathological information. Therefore, relying on single-modal data may have limited the model’s generalizability across different imaging protocols.

An additional limitation of this study is that the datasets were partitioned at the slice level rather than the patient level. Consequently, slices from the same patient may have been distributed across the training, validation, and testing sets, potentially leading to optimistic performance estimates. Although the same data partitioning protocol was consistently applied to all compared models to ensure a fair and unbiased comparison, future work will adopt patient-wise data splitting together with external validation on independent datasets to provide a more rigorous assessment of clinical generalizability.

Moreover, even though the model showed strong performance in segmentation results despite the absence of typical data augmentation (i.e., rotation, flipping, and/or scaling), which showed some of the inherent robustness of the model, not using data augmentation could affect the model’s performance on more diverse datasets, especially those datasets that could contain more variability in tumors and imaging features.

Although the model demonstrated consistent performance across the two evaluated datasets, additional validation using independent multi-institutional datasets acquired under different imaging protocols is still required to further assess its generalizability. Other avenues of interest include expanding the architecture to localize inputs with modality-specific and multi-modal MRI, incorporating a breadth of modern data augmentation techniques for training, and exploring possibilities for the model to be applied to a wide range of medical segmentation tasks other than neuroimaging. These future research directions are consistent with current trends in the literature, where recent brain tumor segmentation studies increasingly emphasize external validation on multi-center datasets, patient-wise evaluation protocols, and multimodal MRI analysis to improve the clinical applicability and generalizability of deep learning models.

Three separate runs (mean ± std) were carried out for every model on the Figshare dataset due to the high computational expense (≈ 6 h per run on a Tesla V100 GPU) and the short revision deadline; single-run results are presented for the TCIA-LGG dataset. Future work will include repeated experiments on the TCIA-LGG dataset to further assess the stability and reproducibility of the proposed model.

## Conclusion

In this work, we have outlined a dual-stream framework, W-AGRU-Net, that tackles some of the challenges in brain imaging analysis through the integration of attention mechanisms, residual learning, and a multi-scale feature extraction paradigm. Our proposed architecture also highlights the role of computational intelligence in improving medical imaging analysis through high-performance segmentation while maintaining computational efficiency in support of deployment into clinical contexts.

The methodological assessment and evaluation of W-AGRU-Net demonstrate an ability to process complex neuroimaging data, showing impressive results on various datasets: (i) **97.87%** Dice and **97.44%** Jaccard on Figshare and (ii) **94.0%** Dice and **89.29%** Jaccard on TCIA-LGG. The computational efficiency of our model output is also evident in a 67% faster inference time than a standard U-Net, bringing to focus its viability for clinical integration. Inference speed in a simulation study has the potential to improve clinical workflows, particularly when accuracy and speed are both important.

This work adds significant value to the area of brain imaging by illustrating and demonstrating how neural architectures of significance can operate on medical images with complex information to extract meaningful information across modalities, and, in doing so, bridge together artificial intelligence and clinical neuroscience. Future studies will build on this visual computing framework on multimodal neuroimaging data and investigate the model’s expansion for other neurological conditions, ultimately contributing new work that operationalizes an expanding intersection of computing and brain science.

## Data Availability

The datasets analysed during the current study are available in the following repositories: Figshare dataset: [https://figshare.com/articles/dataset/brain_tumor_dataset/1512427]TCIA-LGG dataset: [https://www.cancerimagingarchive.net/collection/tcga-lgg/]
